# The effects of prebiotic, probiotic or synbiotic supplementation on overweight/obesity indicators: an umbrella review of the trials’ meta-analyses

**DOI:** 10.3389/fendo.2024.1277921

**Published:** 2024-03-20

**Authors:** Niloufar Rasaei, Mohammadreza Heidari, Fataneh Esmaeili, Sepehr Khosravi, Maryam Baeeri, Ozra Tabatabaei-Malazy, Solaleh Emamgholipour

**Affiliations:** ^1^ Department of Community Nutrition, School of Nutritional Sciences and Dietetics, Tehran University of Medical Sciences (TUMS), Tehran, Iran; ^2^ Network of Interdisciplinarity in Neonates and Infants (NINI), Universal Scientific Education and Research Network (USERN), Tehran, Iran; ^3^ Student Research Committee, Alborz University of Medical Sciences, Karaj, Iran; ^4^ Endocrinology and Metabolism Research Center, Endocrinology and Metabolism Clinical Sciences Institute, Tehran University of Medical Sciences, Tehran, Iran; ^5^ Department of Clinical Biochemistry, School of Medicine, Tehran University of Medical Sciences, Tehran, Iran; ^6^ Non-Communicable Diseases Research Center, Endocrinology and Metabolism Population Sciences Institute, Tehran University of Medical Sciences, Tehran, Iran; ^7^ Toxicology and Diseases Group (TDG), Pharmaceutical Sciences Research Center (PSRC), The Institute of Pharmaceutical Sciences (TIPS), Tehran University of Medical Sciences (TUMS), Tehran, Iran; ^8^ Metabolic Disorders Research Center, Endocrinology and Metabolism Molecular-Cellular Sciences Institute, Tehran University of Medical Sciences, Tehran, Iran

**Keywords:** overweight, obesity, prebiotics, probiotics, synbiotics, meta-analysis

## Abstract

**Background:**

There is controversial data on the effects of prebiotic, probiotic, or synbiotic supplementations on overweight/obesity indicators. Thus, we aimed to clarify this role of biotics through an umbrella review of the trials’ meta-analyses.

**Methods:**

All meta-analyses of the clinical trials conducted on the impact of biotics on overweight/obesity indicators in general populations, pregnant women, and infants published until June 2023 in PubMed, Web of Sciences, Scopus, Embase, and Cochrane Library web databases included. The meta-analysis of observational and systematic review studies without meta-analysis were excluded. We reported the results by implementing the Preferred Reporting Items for Systematic Reviews and Meta-Analysis (PRISMA) flowchart. The Assessment of Multiple Systematic Reviews-2 (AMSTAR2) and Grading of Recommendations Assessment, Development, and Evaluation (GRADE) systems were used to assess the methodological quality and quality of evidence.

**Results:**

Overall, 97 meta-analysis studies were included. Most studies were conducted on the effect of probiotics in both genders. Consumption of prebiotic: 8-66 g/day, probiotic: 10^4^ -1.35×10^15^ colony-forming unit (CFU)/day, and synbiotic: 10^6^-1.5×10^11^ CFU/day and 0.5-300 g/day for 2 to 104 weeks showed a favorable effect on the overweight/obesity indicators. Moreover, an inverse association was observed between biotics consumption and overweight/obesity risk in adults in most of the studies. Biotics did not show any beneficial effect on weight and body mass index (BMI) in pregnant women by 6.6×10^5^-10^10^ CFU/day of probiotics during 1-25 weeks and 1×10^9^-112.5×10^9^ CFU/capsule of synbiotics during 4-8 weeks. The effect of biotics on weight and BMI in infants is predominantly non-significant. Prebiotics and probiotics used in infancy were from 0.15 to 0.8 g/dL and 2×10^6^-6×10^9^ CFU/day for 2-24 weeks, respectively.

**Conclusion:**

It seems biotics consumption can result in favorable impacts on some anthropometric indices of overweight/obesity (body weight, BMI, waist circumference) in the general population, without any significant effects on birth weight or weight gain during pregnancy and infancy. So, it is recommended to intake the biotics as complementary medications for reducing anthropometric indices of overweight/obese adults. However, more well-designed trials are needed to elucidate the anti-obesity effects of specific strains of probiotics.

## 1 Introduction

Over the last four decades, there has been a threefold acceleration in the global prevalence of obesity (1). In 2019, a systematic review and meta-analysis highlighted that 21.4% of elderly individuals in Iran were affected by obesity (2). As a global public health issue, it is linked to the prevalence of different chronic severe conditions, including diabetes, cardiometabolic diseases, hypertension, hyperlipidemia, and malignancy (3, 4). Although the pathogenesis of obesity and overweight is influenced by genetic and environmental factors, it is widely recognized that the primary cause of weight gain is a persistent imbalance between excessive energy intake and inadequate energy expenditure (5–7).

Despite various weight loss strategies being proposed, their long-term effectiveness has been limited. Consequently, there is an increasing demand for innovative methods to supplement existing strategy. The gut microbiota has recently emerged as a critical environmental factor in the development of obesity and its associated metabolic irregularities. Integrating an understanding of gut microbiota with traditional measures, such as a balanced diet and lifestyle modifications, is now recommended for effective weight management (8–12). The gut microbiome, a diverse microbial community in the human digestive system, plays a crucial role in shaping the host’s overall physiology by participating in various metabolic functions (13).

The International Scientific Association of Probiotics and Prebiotics (ISAPP) defines probiotics as live microorganisms offering health benefits upon ingesting in specific quantities. Similarly, prebiotics are characterized as substrates specifically utilized by microorganisms within the host, leading to health benefits. Additionally, synbiotics entail a combination of live microorganisms and substrates that are selectively utilized by microorganisms within the host, resulting in health benefits for the host (14–16). Probiotic and synbiotic supplementation have attracted attention for their potential in regulating gut microbiota and body weight. They can produce short-chain fatty acids that influence hormones responsible for appetite regulation and enhance the resting energy (17–19).

There is accumulating evidence that individuals who are overweight or obese exhibit a distinct profile of the gut microbiota, including reduced microbial gene richness and diversity (known as dysbiosis) compared with normal weight (10, 20, 21). These alterations have been linked to low-grade inflammation, impaired energy metabolism homeostasis, elevated body weight, and dysregulation of insulin signaling (22). Hence, targeting gut microbiota has recently been a promising strategy for treating obesity and related metabolic disorders. Controversy exists regarding the impact of prebiotic, probiotic, or synbiotic consumption on body weight change during gestational diabetes and pregnancy (23, 24), as well as infancy and toddler stages (25–27).

To this point, some studies indicated that supplementing infant formula with prebiotics for full-term infants increases weight gain. Additionally, toddlers consuming milk with synbiotics demonstrated improved growth and greater weight gain (25, 27). However, another systematic review reported that infant formula intake enriched with probiotics or synbiotics did not impact weight in infants and toddlers (26). A meta-analysis showed that probiotic and synbiotic supplements can improve newborn weight among gestational diabetes mellitus patients (23). However, another meta-analysis revealed no significant difference in mean weight at the end of the trial and in gestational weight gain between the intervention group and the control group (24).

Numerous systematic reviews with meta-analysis, though yielding conflicting results, have been conducted to assess the effects of biotics on anthropometric indices such as weight, body mass index (BMI), and waist circumference (WC) (9, 22, 25–29). Moreover, no review of the meta-analysis studies from these trials has comprehensively examined the effect of biotics on various obesity indices in both adults and infants. Some umbrella reviews were conducted in this regard (30–32). However, considering the divergent results in the existing literature, our study extensively searched all interventions to attain a more comprehensive understanding. This umbrella review of the trials’ meta-analysis studies aims to give a snapshot of the influence of prebiotic, probiotic, or synbiotic intake on body weight changes, irrespective of age and sex differences.

## 2 Methods and materials

This study adhered to the Preferred Reporting Items for Systematic Reviews and Meta-Analysis (PRISMA), ensuring the reporting of preferred items for systematic reviews and meta-analyses (33).

### 2.1 Search strategy

A comprehensive search was conducted across multiple international web databases, including PubMed, Scopus, Web of Science, Embase, and Cochrane Library, to identify pertinent meta-analyses exploring the relationship between prebiotic, probiotic, or synbiotic supplementation and body weight. This search encompassed records up to June 2023. Furthermore, the bibliographies of research papers were examined to identify potential studies that may have been overlooked during the initial search. The PICO (Participant, Intervention, Comparison/Control, Outcome) search framework was employed to systematically explore articles on the effects of prebiotic, probiotic, or synbiotic supplementation on overweight/obesity indicators such as body weight gain, BMI, or WC. An English language criterion was applied for inclusion to ensure comprehensive coverage of relevant studies. To prevent the omission of pertinent research, a combination of MeSH terms and keywords was employed as the initial approach for data collection. Our exclusion criteria were meta-analyses of observational studies, in vivo/in vitro research, case reports, and systematic review studies without meta-analysis. Gray literature and conference abstracts were considered if they provided substantial data. Hand-searching of the reference list of the included studies was performed to find relevant studies. A comprehensive outline of the search strategy is available in **Supplementary Table S1**.

### 2.2 Study selection

Research studies were deemed eligible for inclusion if they met most of the following criteria: (1) Systematic review/meta-analysis of various types of clinical trials; (2) examined the sole or combination intake of prebiotic, probiotic, or synbiotic; (3) evaluated the impact of prebiotic, probiotic, or synbiotic supplementation on overweight/obesity indicators such as weight gain, BMI or WC; (4) compared the effects of the supplementation with either a placebo or a standard treatment as the control group; (5) encompassed participants of all age groups and genders; (6) included healthy individuals, or those with any medical condition.

### 2.3 Data extraction

FE and MRH independently assessed the validity of eligible studies by reviewing titles and abstracts, extracting outcomes, and evaluating the credibility of the included publications. Consensus was achieved through consultation with the corresponding authors (OTM and SE) in the discrepancies. Data points encompassed title, authors, publication year, geographic region of the study, population details (total number, age, gender, underlying condition), the number and design of included trials, intervention dose and duration, primary outcome, subgroup analyses, dose-response findings, and reported effective dosage. Three e-mails were frequently sent to the corresponding author to find the full text of inaccessible studies.

### 2.4 Quality assessment

FE and MRH independently assessed the quality of the studies, addressing discrepancies through consultation with OTM and SE. The evaluation employed the “A Measurement Tool to Assess Multiple Systematic Reviews–2” (AMSTAR2), a validated tool suitable for assessing the internal validity of intervention-focused systematic reviews (34). The evaluation results were tabulated in **Supplementary Table S2**. Moreover, The Grading of Recommendations Assessment, Development, and Evaluation (GRADE) systems were used to assess the evidence quality (35), (**Supplementary Table S3** and **Table 1**). Rating discrepancies were discussed and resolved, involving a third party if needed.

## 3 Results

### 3.1 Studies characteristics

**Figure 1:**
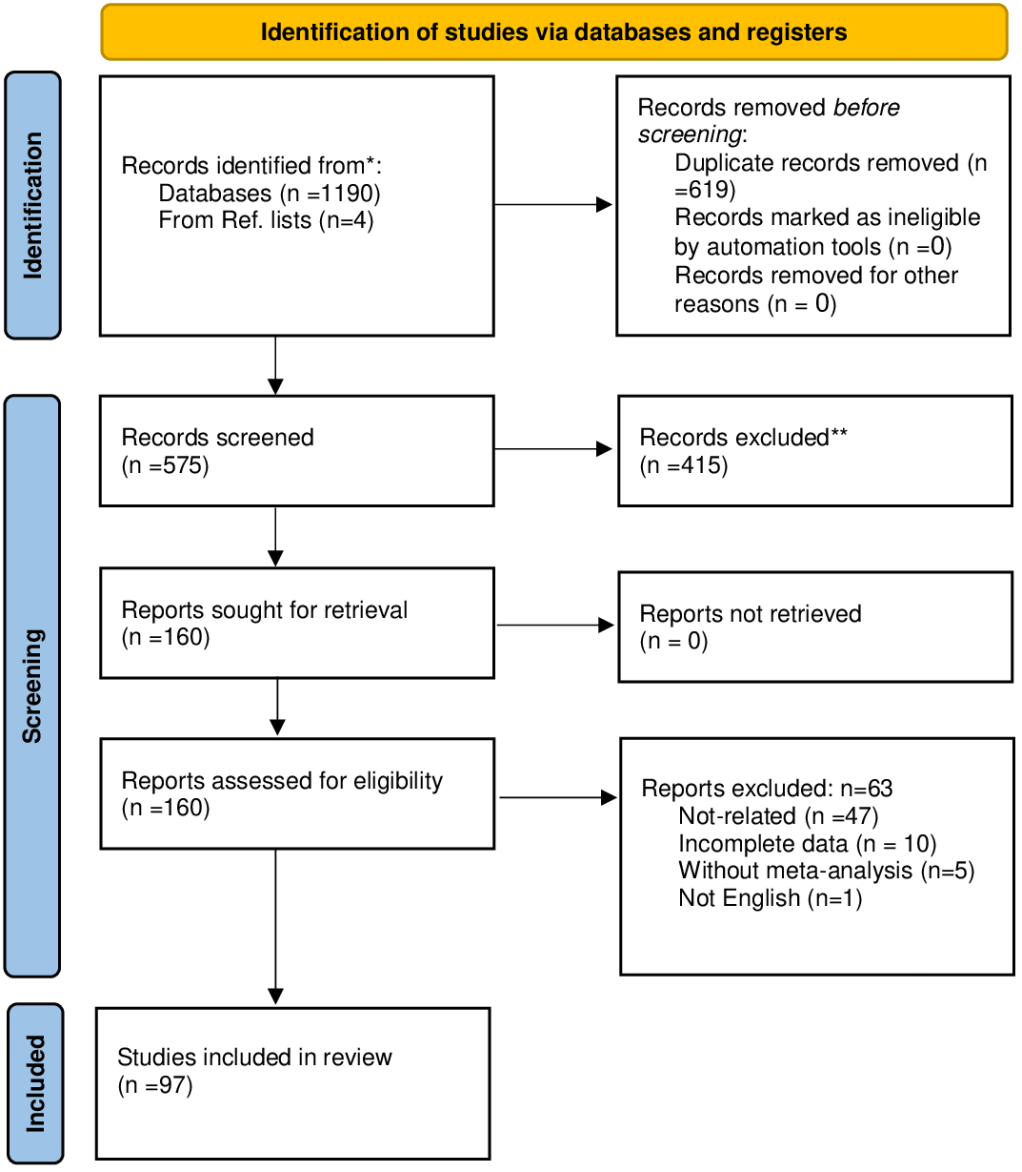
PRISMA flow diagram of the study process.

A total of 97 papers satisfied the inclusion criteria and were incorporated into the study. The search process for these studies under the PRISMA flowchart is outlined in **Figure 1**. Subsequently, based on the population studied, they were classified into three distinct groups: (1) general population, (2) pregnant, and (3) infants. Comprehensive details of the selected studies can be found in **Supplementary Table S3** and **Table 1**.

**Table 1:** Characteristics of included meta−analyses of clinical trials in pregnant women, or infants.

Reference	Design	Study Population	Total (N)	Sex (B, F, M)	Age (mean, range(y))	IncludedStudies (N)	Intervention/control	Intervention		QualityAssessment(yes/no)	Effects Model	MA metric (MD, WMD, SMD)	MA Outcomes			Heterogeneity		GRADElevel
								dose (mg, g, CFU,…)	duration (w)				Estimates	95% CI	p Value	I^2^ (%)	p Value	
GDM																		
Weight gain pregnancy																		
Chatzakis et al, 2019 (36)	RCT	GDM in overweight/ obese pregnant women	4237	F	NR	5	Probiotics vs Placebo	NR	11w of gestation - 6w postartum	yes	Random	NR	Direct MA: 0.2	-0.69, 1.10	Non-sig.	43	NR	⨁⨁⨁◯ Moderate
											network meta−analysis		Network MA: 0.5	-10.9, 11.9	Non-sig.	NR	NR	⨁⨁⨁◯ Moderate
Pan et al. 2019 (37)	RCT	GDM	830	F	28- 34	6	Probiotic/control	2×10^9^-112.5×10^9^	6-12	yes	Fixed	SMD	-0.11	-0.38, 0.16	0.43	0	0.46	⨁⨁⨁⨁ High
Okesene-Gafa et al. 2020 (38)	RCT	GDM	695	F	18-49	9	Probiotic/placebo	2× 10^9^ CFU	6-19	yes	Fixed	MD	1.38	-0.49, 3.24	0.15	0	0.75	⨁⨁⨁⨁ High
			177	NR	infants	4						Total gestational weight gain, MD	0.24	-0.3, 0.78	0.38	0	0.48	⨁⨁⨁⨁ High
Zhou et al 2021 (39)	RCT	GDM	894	F	26- 34	12	Probiotic or synbiotic/placebo	NR	4- 8	yes	Fixed	SMD	-0.04	-0.15, 0.07	0.51	31	NR	⨁⨁⨁⨁ High
Chu et al., 2022 (40)	RCT	GDM in overweight or obese pregnant women	1048	F	28- 32	5	Probiotic/placebo	10^9^ – 10^10^ CFU/d	4 -24 post partum	yes	Random	RR	0.92	0.79, 1.06	0.223	91.2	0.001	⨁⨁⨁⨁ High
Mu et al., 2023 (41)	RCT	GDM	390/389	F	26-34	11	Probiotics/synbiotics placebo	1 × 10^9^ CFU/capsule -112.5 × 10^9^	4-8	yes	Random	Gestational weight gain, MD	0.09	-0.08, 0.26	0.29	0	0.64	⨁⨁⨁◯ Moderate
Yefet et al., 2023 (42)	RCT	GDM	430/424	F	NR	14	Probiotic/placebo	10^6^ - 112.5 × 10^9^	4w and until delivery	yes	Random	MD	-0.008	−0.113, 0.097	Non-sig.	8.46	0.361	⨁⨁⨁◯ Moderate
Mother BMI change																		
Zhou et al., 2021 (39)	RCT	GDM	894	F	26- 34	12	Probiotic or synbiotic/placebo	NR	4- 8	yes	Fixed	SMD	0.06	-0.02, 0.14	0.14	0	NR	⨁⨁⨁⨁ High
Birth Weight																		
Chatzakis et al., 2019 (36)	RCT	GDM in overweight/ obese pregnant women	4237	F	NR	5	Probiotics vs Placebo	NR	11w of gestation - 6w postartum	yes	Random	NR	Direct MA: 0.06	-0.06, 0.19	Non-sig.	5	NR	⨁⨁⨁◯ Moderate
											network meta−analysis		Network MA: 0.06	-0.06, 0.19	Non-sig.	NR	NR	⨁⨁⨁◯ Moderate
Okesene-Gafa et al., 2020 (38)	RCT	GDM	177	NR	infants	4	Probiotic/placebo	2× 10^9^ CFU	6-19	yes	Fixed	Birth weight	-79.14	-183.0, 24.73	0.93	0	0.59	⨁⨁⨁⨁ High
Wang et al, 2020 (43)	RCT	GDM or Overweight/Obesity	540/553	F	18-45	7	Probiotic/placebo	10^9^ CFU/g to 6.5 × 10^9^ CFU/g /d	4, 6 wk or from eNRollment to birth	yes	Random	MD	-10.270	-90.17, 69.63	0.801	33.955	0.169	⨁⨁⨁⨁ High
Zhou et al 2021 (39)	RCT	GDM	894	F	26- 34	12	Probiotic or synbiotic/placebo	NR	4- 8	yes	Fixed	SMD	-0.29	-0.5, -0.09	0.006	17	NR	⨁⨁⨁⨁ High
Chu et al., 2022 (40)	RCT	GDM in overweight or obese pregnant women	1048	F	28- 32	5	Probiotic/placebo	10^9^ – 10^10^ CFU/d	4 -24 post partum	yes	Random	WMD	28.47	-34.8, 91.73	0.383	4.5	0.381	⨁⨁⨁⨁ High
Yefet et al 2023 (42)	RCT	GDM	430/424	F	NR	14	Probiotic/placebo	10^6^ - 112.5 × 10^9^	4w and until delivery	yes	Random	MD	-93.25	−200.43, 13.93	Non-sig.	33.07	0.176	⨁⨁⨁◯ Moderate
Pregnancy																		
**Maternal weight change**																		
Han et al 2018 (44)	DBRCT	Pregnancy	1139	F	18–40	10	Probiotic/placebo	10^7^- 10^10^ CFU/g	4–24	yes	Random	MD	-0.27	-0.61, 0.08	0.13	87	<0.0001	⨁⨁⨁◯ Moderate
Jarde et al 2019 (45)	RCT	Pregnancy women whose infants at risk of atopy and/or allergies, healthy, GDM	4098	F	NR	21	Probiotic/control	NR	1-25	yes	Random	MD	0.13	-1.98, 2.23	0.91	68	0.05	⨁⨁⨁⨁ High
Davidson et al. 2021 (46)	DBRCTs	Pregnancy with high risk of GDM	1244	F	>18	7	Probiotics/placebo	1-10 billion CFU/g	4-24	yes	Random	MD	0.3	-0.67, 1.26	0.54	40	0.17	⨁⨁⨁⨁ High
Birth weight																		
Vahdaninia et al, 2016 (47)	RCT	Pregnancy	485	F	NR	4	Probiotic/NR	NR	NR	yes	Random	Obesity of children, RR	0.36	0.03, 3.9	Sig.	54.8	NR	⨁⨁⨁⨁ High
												Overweight in children, RR	0.73	0.4, 1.36	Sig.	0	NR	⨁⨁⨁⨁ High
Han et al 2018 (44)	DBRCT	Pregnancy	1139	F	18–40	10	Probiotic/placebo	10^7^ - 10^10^ CFU/g	4–24	yes	Fixed	MD	37.88	-18.32, 94.07	0.19	55	0.06	⨁⨁⨁◯ Moderate
Jarde et al 2019 (45)	RCT	Pregnancy women whose infants at risk of atopy and/or allergies, healthy, GDM	4098	F	NR	21	Probiotics, prebiotics /control	NR	1-25	yes	Random	MD	6.76	-38.52, 52.04	0.77	0	0.69	⨁⨁⨁⨁ High
							Prebiotic/control					MD	-63.95	-262.02, 134.12	0.53	NA	NA	⨁⨁⨁⨁ High
							Probiotic/control		MD			10.66	-35.85, 57.18	0.65	0	0.65	⨁⨁⨁⨁ High
Davidson et al. 2021 (46)	DBRCTs	Pregnancy with high risk of GDM	1524	NR	infants (1d-24 mo)	6	Probiotics/placebo	1-10 billion CFU/g	4 -24	yes	Random	MD	26.87	-49.52, 103.26	0.49	42	0.12	⨁⨁⨁⨁ High
Pérez−Castillo et al 2021 (48)	RCT	Pregnant women (healthy, obese/overweight, GDM)	8519	F	NR	25	Probiotic/control	5 × 10^5^ CFU to 5 × 10^10^ CFU	3 -26	yes	Random	MD	-5.36	-37.6, 26.89	0.74	0	0.5	⨁⨁⨁⨁ High
Infancy																		
Weight gain																		
Rao et al 2009 (49)	RCT	Full−term neonates	1459	NR	2wk, 26 wk	11	Prebiotic/control	0.15 - 0.8 g/dL	2 -24	yes	Fixed	WMD	1.07	0.14, 1.99	0.2	0	0.62	⨁⨁⨁⨁ High
Steenhout et al 2009 (50)	DBRCT	Infants	329	B	NR	5	Probiotic (Bifidobacterium lactis)/control	2×10^7^- 3×10^7^	17.14	no	Random	MD	1.5	0.09, 2.93	0.0368	NR	NR	⨁⨁⨁⨁ High
Szajewska et al 2013 (51)	RCT	Healthy infant	472	NR	6d, 7 wks	7	Bifidobacterium lactis/control	10^6^ - 3.6 × 10^9^ CFU/ 1 g of formula	4-28	yes	Random	MD	0.96	-0.7, 2.63	0.26	31.7	0.22	⨁⨁⨁◯ Moderate
											Fixed	0.9	-0.51, 2.32	Non-sig.	54.1	NR	◯⨁⨁⨁ Moderate
Sun et al 2017 (52)	RCT	Very preterm infants	4,496/4,452	B	NR	32	Probiotic/control	6.6 × 10^5-^6 × 10^9^	4-6	yes	Random	MD	-0.29	-1.16, 0.58	0.51	0	0.97	⨁⨁⨁⨁ High
Monicaasun 2022 (53)	RCT	Infants	1176	NR	NR	6	Probiotics/ control	NR	12	yes	Fixed	SMD	-1.05	-1.25, -0.84	p < 0.00001	98	p < 0.00001	⨁⨁⨁⨁ High
Panchal et al 2022 (54)	RCT	Preterm infants	4817	NR	NR	30	Probiotic/placebo	2 million to 10 billion CFU/d	3- 6	yes	Random	SMD	0.24	0.04, 0.44	0.02	88	0.00001	⨁◯◯◯ Very low
Janmohammadi et al. 2023 (55)	RCT	Infants	1554	B	1 d-12 mo	11	Synbiotics/ control	NR	4- 52	yes	Random	WMD	2.06	-4.08, 8.21	0.51	99.4	0	⨁◯◯◯ Very low
BMI																		
Steenhout et al 2009 (50)	DBRCT	Infants	329	B	NR	5	Probiotic (Bifidobacterium lactis)/control	2×10^7^- 3×10^7^	17.14	no	Random	MD	3.44	0.13, 6.75	0.0418	NR	NR	⨁⨁⨁⨁ High
Szajewska et al 2013 (51)	RCT	Healthy infant	472	NR	6d, 7 wks	7	Bifidobacterium lactis/control	10^6^ - 3.6 × 10^9^ CFU per 1 gram of formula	4-28	yes	Fixed	MD	0.09	-0.05, 0.22	0.21	0	0.64	⨁⨁⨁◯ Moderate

B, Both; F, Female; M, Male; ROB, Risk of Bias; NR, Not reported; MA, meta−analysis; RCTs, Randomized controlled trials; DBRCTs, double−blind controlled trials; SCBRTs, single−blind controlled trials, WMD, Weighted mean difference; SMD, Standardized mean difference; ASMRT, A MeaSurement Tool to Assess systematic Reviews; T2DM, type 2 diabetes mellitus; RR, Relative risk; GDM, gestational diabetes mellitus; BW, body weight; BF, body fat; NA, Not applicable; GRADE, Grading of Recommendations Assessment, Development, and Evaluation.

Most included studies were meta-analyses of clinical trials centered on prebiotic, probiotic, or synbiotic intervention. However, one meta-analysis study focused on clinical trials besides observational studies exploring probiotic supplementation. These studies encompassed both genders and examined the effects of the biotics versus placebo.

The study populations ranged from 134 to 1,324,640 participants, spanning ages from 1 day to 87 years. Participants comprised healthy individuals as well as patients with various background conditions, including metabolic syndrome (MetS), type 2 diabetes mellitus (T2DM), dyslipidemia, obesity, and hypertension (HTN). Almost all of the included meta-analyses underwent quality assessment. Further details of the included studies are provided as follows.

### 3.2 Effects of prebiotic, probiotic, or synbiotic supplementation on overweight/obesity measurements in the general population

Overall, sixteen papers assessed the prebiotic effect on overweight/obesity variables. Among these trials, the most frequently studied conditions were non-alcoholic fatty liver disease (NAFLD) or obesity. The primary outcome measures in these studies were focused on body weight (56–63), BMI (56, 57, 59, 61–66), WC (62, 64, 67, 68), or body fat mass (BFM) (56, 57, 62). Prebiotic supplementation exhibited a significant reducing effect on body weight and BMI. Nonetheless, the impact of prebiotic consumption on body weight and BMI among patients with NAFLD and DM displayed inconsistency (64). Regarding body weight, 3 out of 5 studies demonstrated a weight-reducing effect (56, 58, 67). In the case of BMI, a reducing effect was observed in 2 out of 5 studies (65, 67). Similarly, investigations into the effects of prebiotic supplementation on BFM (56, 57) and WC (65, 67) showed a neutral effect on these measurements. The participants’ ages spanned from 1 day to 77 years, and both genders were represented. The administered doses and duration of the studies ranged from 0.007 × 10^9^-150 ×10^9^ colony-forming unit (CFU)/day to 0.88-66000 mg/d for 1-104 weeks. Notably, significant body weight and BMI reductions were observed with prebiotic treatment lasting more than 7 and 15 days, utilizing 8- 66 gr/day doses for both parameters.

A total of sixty-two papers were incorporated in assessing probiotics’ effect on obesity variables. All of them were clinical trial meta-analyses, and an additional one focused on clinical trials and observational studies. The research primarily concentrated on obese individuals with NAFLD aged one day to 85 years old. The main measured indicators were body weight (23, 28, 56, 59, 69–85), BMI (23, 28, 56, 59, 60, 65, 69–82, 84–105), WC (28, 68, 70, 72, 75, 79–81, 85, 87, 90, 93, 94, 99, 101), hip circumference (HC) (90), waist to hip ratio (WHR) (78, 80, 90, 94), body fat percent (BF%) (71, 75, 80, 85, 90, 94), and BFM (56, 61, 71, 72, 75, 80, 81, 90). Probiotic supplementation significantly reduced weight, BMI, WC, BF%, and BFM across most studies. However, investigations into the impact of probiotic intake on variables such as HC (90) and percentage of excess weight loss (%EWL) (76, 105) revealed neutral effects on these measurements. In summary, the dosages of probiotic supplements varied widely among the included studies in 1.0 × 10^4^ to 1.35 × 10^15^ CFU/day, with administration durations ranging from 2 to 104 weeks. Notably, body weight and BMI showed significant reductions with probiotic treatment for over 15 days at doses ranging from 4.97 × 10^6^ to 7 × 10^11^ CFU/day.

Also, twenty-five meta-analyses of clinical trials were included in the current study to examine the impact of synbiotics on overweight/obesity indices. Their main focus of the outcome measurements was on body weight (24, 28, 56, 106), BMI (28, 56, 91, 106, 107), WC (28, 68, 106, 107), and BFM (56). Synbiotic supplementation exhibited notable decreasing effects on WC in 4 out of 6 studies (68, 106–108), as well as on BFM in 1 out of 2 studies (109). However, the impact of synbiotic consumption on weight and BMI showed inconsistency. Among the included studies, 3 out of 7 reported a reduction in body weight (63, 106, 107), and 2 out of 6 studies observed a decrease in BMI (63, 91) following synbiotic supplementation. The study participants, spanning both genders and aged from 1 day to 85 years old, received varied treatment doses of synbiotics: 0.5-300 g/day and 10^6^-1.5×10^11^ CFU/day over durations ranging from 2 to 104 weeks. Synbiotic treatment for more than 15 days and at doses ranging from 3.7 × 10^6^ to 1 × 10^11^ CFU/day and 9-150 g/d led to a reduction in body weight. In addition, in some studies, the effect of consuming prebiotics, probiotics, and synbiotics was not reported separately, and their collective impact was stated in comparison with the control group. BMI (56, 110) and weight reduction (56, 111) were observed in some studies, but a neutral effect on BMI (112) and EWL% (113) was found in others.

### 3.3 Effects of prebiotic, probiotic, or synbiotic supplementation on overweight/obesity measurements in pregnancy population

The prebiotic, probiotic, or synbiotic did not have any beneficial effects during pregnancy on weight and BMI with treatment doses 6.6 × 10^5^-10^10^ CFU/day of probiotics during 1-25 weeks and 1 × 10^9^-112.5 × 10^9^ CFU/capsule of synbiotic during 4-8 weeks. The effect of prebiotics on these anthropometric indices during pregnancy was assessed in only one study (114) in which the administered doses were not reported. The duration of prebiotic usage during pregnancy varied, ranging from 1 to 25 weeks, and was administered in differing doses. Vahdaninia et al. (115) showed a significant increase in overweight/obesity in children with probiotic supplementation in pregnant women (116). In addition, probiotic or synbiotic supplementation compared to placebo consumption (23) showed a negative significant effect on newborn weight in GDM with a neutral impact on gestational weight and BMI change, as well as mother weight and BMI at the end of the trial. In the rest of the included studies, there was no prebiotic, probiotic, or synbiotic supplementation effect on birth weight, gestational weight gain, and weight change for mothers during pregnancy.

### 3.4 Effects of prebiotic, probiotic, or synbiotic supplementation on overweight/obesity measurements in infants

The dosage administered for prebiotics and probiotics in infancy were 0.15 to 0.8 g/dL (2-24 weeks) and 2×10^6^ -6 × 10^9^ CFU/day (2-24 weeks), respectively. Synbiotics were used in infancy for ages 4 to 52 weeks, with doses varying across the studies. Overall, one study assessed the effects of prebiotic supplementation on weight gain (117), which showed a non-significant increase. Also, the consumption of probiotics observed a non-significant impact on weight gain, except in two studies (118, 119), which reported a reduction in weight gain, and another study (119), showed an increase in weight gain for a short term. Moreover, only one study (120), investigated the effect of synbiotics on weight gain, and the results were neutral.

### 3.5 Quality of methodology and evidence

The AMSTAR2 assessment revealed that 23 studies were categorized as having critically low quality, while 24 were rated as low quality. The primary limitations in these assessments were associated with item two, which concerns the registration protocol before conducting the review; item seven, which involves providing a list of excluded studies and justifying their exclusions; and item 10, which pertains to the reporting of funding sources for individual studies (refer to **Supplementary Table S2** for details).

Among the six outcomes investigated in the general population, a significant proportion of studies that examined the impact on BMI (54.76%) or weight (53.06%) found evidence of moderate or high quality, with estimated significant positive effects on reducing BMI or weight. Studies focusing on weight predominantly exhibited moderate to high-quality evidence (53.06%). However, the quality of evidence for the three studies concerning the percentage of excess weight loss was notably low, and they did not show a significant reduction in excess weight among obese and morbidly obese participants.

For studies related to WC, the majority (70%) demonstrated high or moderate-quality evidence, indicating significant beneficial effects. In contrast, studies investigating HC and WHR were characterized by low-quality evidence in 83.3% of cases and did not show significant reductions in HC or WHR. Moreover, most studies examining the impact on BFM (62.5%) were backed by either high or moderate-quality evidence, and these studies revealed significant enhancements, as outlined in **Supplementary Table S3**.

In the context of GDM, pregnancy, and infancy populations, when considering the seven outcomes, 66.67% of the studies exhibited high-quality evidence, 27.78% were associated with moderate-quality evidence, and a mere 5.56% had very low-quality evidence, as indicated in **Table 1**. However, when assessing the impact of probiotics, prebiotics, or synbiotics compared to control or placebo on maternal or infant weight gain and BMI, the overall findings were non-significant for patients with GDM, pregnant women, and infants.

## 4 Discussion

Our study revealed that the prebiotics showed favorable impacts on body weight and BMI reduction across diverse populations beyond 15 days and by doses of 0.88-66 g/day, with any significant reduction of WC and BFM in adults. However, the probiotics at doses ranging from 104 to 1.35×1015 CFU/day for durations exceeding 7 and 15 days, respectively, decreased body weight and BMI. Moreover, the synbiotic had a favorable impact on weight, BMI, WC, and BFM at doses of 10^6^-1.5×10^11^ CFU/day and 0.5-300 g/day for over 15 days. The prebiotic and probiotic had a neutral effect on weight gain and weight change in pregnant women by doses 6.6 × 10^5^-10^10^ CFU/day of probiotics during 1-25 weeks’ treatment. The effect of biotics on weight and BMI in infants was mostly non-significant, except in two studies in which probiotics (2×10^6^ -6 × 10^9^ CFU/day for 2-24 weeks had significant but opposite effects on weight gain. Despite negatively affecting newborn weight, synbiotic supplementation in individuals with gestational diabetes mellitus (GDM) did not significantly influence weight gain, weight change, BMI change, or maternal weight and BMI at the end of the trial.

Prebiotics, probiotics, and synbiotics have shown potential anti-obesity effects through various mechanisms. Prebiotics are non-digestible fibers that serve as food for beneficial gut bacteria such as *Bifidobacteria* and *Lactobacilli*. By selectively promoting the growth of these bacteria, prebiotics contribute to a healthier gut microbiota composition. Dewulf et al. found that prebiotics prompt an increase in the proportions of *Bifidobacterium* and *Faecalibacterium* in the gut microbiota while reducing Body Fat Mass (BFM) in obese women (121). These beneficial bacteria produce short-chain fatty acids (SCFAs) during the fermentation of prebiotics. SCFAs have been shown to have positive effects on metabolism and inflammation. They can regulate appetite and promote the utilization of energy from food, potentially helping to prevent excess calorie storage. SCFAs interact with receptors on epithelial cells within the gut lining, elevating levels of glucagon-like peptide 1 (GLP-1) and peptide YY (PYY), thereby enhancing satiety (122, 123). A study using 21 g/d doses of inulin revealed increased PYY, GLP-1, leptin, satiety, reduced ghrelin, energy intake, body weight, and BFM (124, 125). Furthermore, prebiotic treatment upregulates peroxisome proliferator-activated receptor (PPAR)γ and PPARα expression (125) while concurrently downregulating sterol regulatory element-binding protein-1c (SREBP-1c) and fatty acid synthase expression, thus diminishing fatty acid production. This suggests that prebiotics may positively impact lipid metabolism by influencing gene expression (126). Additionally, prebiotics, which are viscous plant-derived oligosaccharides, have been found to delay gastric emptying and enhance feelings of satiety. This effect is attributed to their high soluble fiber content, which slows down the movement of food through the digestive tract. They can also interfere with dietary cholesterol uptake and bile acid reabsorption, leading to beneficial effects on lipid metabolism (127, 128).

Biotic supplementation may also influence the gut-brain axis, a bidirectional communication system between the gut and the brain. Some studies suggest they can modulate hormones such as ghrelin and peptide YY, which control hunger and satiety. Improved gut barrier function is another mechanism for the beneficial effects of biotics. Dysbiosis can weaken the gut barrier and contribute to metabolic disorders. Prebiotics support the growth of beneficial bacteria, which can enhance the integrity of the gut barrier. This reduces the absorption of endotoxins and other potentially harmful molecules, which could play a role in reducing inflammation and obesity-related complications (129). Obesity is linked to chronic low-grade inflammation, which contributes to metabolic disturbances. Probiotic supplementation can interact with the immune system, influencing the production of inflammatory cytokines. By fostering an anti-inflammatory environment both in the gut and systemically, specific probiotics may assist in reducing inflammation and enhancing insulin sensitivity (130).

One of the main challenges in determining the effectiveness of currently available probiotic preparations for weight control is the presence of different confounding factors (131). Some studies may have implemented relatively short durations, potentially insufficient for significant changes in anthropometric measurements to manifest. Studies conducted over longer terms might produce different outcomes.

Assessing the effectiveness of currently available probiotic preparations for weight control presents a significant challenge, primarily due to various confounding factors. These factors can include differences in the strains and formulations of probiotics used, variations in individual responses to probiotics, dietary habits, genetic predispositions, lifestyle factors, and personal gut microbiota composition. Additionally, the duration of probiotic supplementation, the specific target population, and the quality of study designs all contribute to the complexity of evaluating their impact on weight management. To draw meaningful conclusions about the effectiveness of probiotics in weight control, it is essential to account for and mitigate these confounding factors in research and analysis.

Here, we can discuss the abovementioned factors based on the available literature. Probiotics from the *Bifidobacterium* and *Lactobacillus genera* have notable effects on weight management. These genera are among the most widely studied for their anti-obesity, anti-inflammatory, and immunomodulatory properties (128, 132). However, changes in BMI remained negligible following intervention with the Probiotic *Lactobacillus rhamnosus* GG, indicating that the probiotic treatment didn’t significantly influence body weight in obese children with hepatic issues (133). Conversely, *Lactobacillus gasseri* BNR17 supplementation reduces visceral fat accumulation and WC in obese adults (134).

Regarding the duration of biotic supplementation, a meta-analysis found that administering synbiotics to infants for 3-52 weeks had no significant impact on weight. In children, supplementation for 8-104 weeks resulted in weight gain, whereas in adults, supplementation over 2-26 weeks led to weight loss (111). These findings imply that the impact of synbiotics on body weight could depend on the type of additive, the duration of the administration, and the host. The effectiveness and safety might differ based on microorganism strains and doses. Another meta-analysis, examining 3-24 weeks of synbiotic intake at doses of 4.97×10^6^-1.5×10^11^, observed reduced waist circumference (WC) without significant weight or BMI changes. This hints at synbiotics’ potential to target abdominal adiposity (28). Supplementation with *Lactobacillus rhamnosus* GG before delivery and for six months postpartum reduced weight gain in 1-4-year-olds, but this effect didn’t persist after a decade (135). While the short-term of probiotics on weight management in children is promising, the long-term intervention is still unclear. Also, according to this umbrella review, probiotic supplementation did not affect gestational weight gain.

Studies investigating the effect of prebiotic, probiotic, or synbiotic consumption on weight changes during various life stages, including pregnancy (23, 24), infancy, and early childhood (25–27), have yielded inconsistent results. Some research indicated increased weight gain in full-term infants fed prebiotic-enriched formula, while others noted improved growth and weight gain in toddlers consuming synbiotic-fortified milk (25, 27). Conversely, supplementing infant formula with probiotics or synbiotics showed no significant weight effect in another systematic review (26). Additionally, a meta-analysis found no noteworthy differences in mean end-of-trial weight or gestational weight gain between intervention and control groups (24). Although, to the best of the author’s knowledge, this umbrella review represents the initial endeavor to comprehensively explore existing meta-analyses in the studied topic, irrespective of age and gender, it should be noted that our findings may not be generalizable to all populations and health conditions. The heterogeneity in the effects of prebiotic, probiotic, and synbiotic supplementation on overweight/obesity indicators arises from intricate interactions among several factors. Firstly, studies examining the effects of the abovementioned intervention on these indices often vary in terms of participant characteristics, intervention protocols, duration of supplementation, and outcome measures.

Diversity can lead to differing results, as the impact of the intervention may depend on the specific context and conditions of each study. Secondly, the composition of gut microbiota and metabolic responses can significantly differ among individuals. Genetic factors, dietary patterns, lifestyle, and pre-existing health conditions all play a role in this variation (135). Consequently, individuals’ responses to each supplementation may differ, leading to inconsistent outcomes across participants. Underlying health conditions like MetS, obesity, or diabetes can influence how the body reacts to supplementation by impacting metabolic pathways and gut microbiota composition, altering expected outcomes (136). Thirdly, variability in the study population, including age, gender, ethnicity, and baseline health status, can contribute to heterogeneous responses to biotic supplementation (136, 137). What works effectively in one population might produce different effects in another. More importantly, the specific strains of probiotics and types of prebiotics used in synbiotic formulations can influence the outcomes. Different strains and types of these components interact distinctly with the gut microbiota and host metabolism, resulting in a range of genus-specific effects on anthropometric measurements. Ensuring standardized viable bacterial cells in commercial probiotics is crucial for research and clinical studies. Accurate dosing allows scientists and healthcare professionals to replicate results and make meaningful comparisons between studies. It also enables more precise insights into the relationship between probiotics and health outcomes.

To accurately evaluate probiotic efficacy across health conditions, study design and analysis must account for these confounding factors. Finally, we should pay attention to the role of epigenetic factors when discussing the inconsistent effects of prebiotics and probiotics on birth weight and childhood weight gain. Epigenetics refers to changes in gene expression that are influenced by factors such as environment, lifestyle, and diet. Epigenetic mechanisms can be crucial in shaping how genes are activated or silenced, impacting various developmental outcomes. The effects of prebiotics and probiotics on birth weight and weight gain might be mediated through epigenetic modifications (138). However, these modifications can be intricate and multifaceted, influenced by genetic and environmental factors. Understanding the complex interplay between prebiotics, probiotics, epigenetics, and growth outcomes requires comprehensive research to decipher the specific epigenetic pathways influenced by these interventions and how they contribute to the observed effects on birth weight and weight gain.

Our study has particular strengths and limitations. The study’s main strength is the overview of the meta-analyses’ trials. As it is known, the meta-analysis of clinical trials is at the highest level of evidence-based medicine (139). The second strength is using standard tools to assess the quality of the methods in the included studies (AMSTAR2) and the strength of the evidence (GRADE). The study’s main limitation is disagreement among various meta-analyses because of variations in multiple factors. These include differences in sample sizes, participants’ health conditions, the quality ratings of studies, the dose of supplements provided, the techniques employed to measure anthropometric indices, the absence of evaluation of confounding variables, and failure to conduct subgroup analysis. These discrepancies contribute to inconsistencies among the meta-analyses.

## 5 Conclusion

In conclusion, this umbrella review highlights the potential role of probiotics as supplementary treatments in managing the anthropometric indices associated with overweight and obesity in adults. Although current findings in the literature are encouraging, they also reveal the complexity inherent in the interactions between the gut microbiota and the host. To fully realize the therapeutic potential of probiotics in this area, upcoming research should focus on enhancing the precision and consistency of study designs, standardizing intervention protocols for better comparison, and identifying the specific probiotic strains with the most effective anti-obesity properties. Furthermore, a deeper investigation into the mechanisms underlying these effects is essential. Advancements in these areas will provide more consistent, reliable, and detailed insights, thereby facilitating the development of more effective and tailored treatment strategies to combat obesity.

## Author contributions

NR: Writing – original draft, Writing – review & editing. MH: Data curation, Methodology, Validation, Writing – review & editing. FE: Data curation, Methodology, Validation, Writing – review & editing. SK: Data curation, Methodology, Writing – review & editing. MB: Data curation, Methodology, Writing – review & editing. OT-M: Conceptualization, Investigation, Methodology, Project administration, Supervision, Validation, Writing – review & editing. SE: Investigation, Supervision, Validation, Writing – review & editing.
