# Supplementary material for: The effects of prebiotic, probiotic or synbiotic supplementation on overweight/obesity indicators: an umbrella review of the trials’ meta-analyses
Source: Front Endocrinol (Lausanne). 2024 Mar 20;15:1277921. doi: 10.3389/fendo.2024.1277921 (PMC10987746; doi:10.3389/fendo.2024.1277921)
Supplement: Supplementary file 1 [file DataSheet_1.pdf]

Table S1-Search strategy in the searched web databases

PubMed

(Probiotic*[TIAB] OR Synbiotic*[TIAB] OR Prebiotic*[TIAB]) AND (Obesity[TIAB] OR obes*[TIAB] OR overweight[TIAB] OR bmi[TIAB] OR weight[TIAB] OR (body[TIAB] AND mass[TIAB] AND index[TIAB]) OR (body[TIAB] AND composition[TIAB]) OR ((waist[TIAB] OR hip[TIAB]) AND circumference[TIAB]) OR (fat[TIAB] AND mass[TIAB])) AND (meta-analysis[TIAB] OR "meta analysis"[TIAB])

EMBASE

#1 Probiotic*:ti,ab OR Synbiotic*:ti,ab OR Prebiotic*:ti,ab

#2 'prebiotic agent'/exp OR 'synbiotic agent'/exp OR 'probiotic agent'/exp

#3 #1 OR #2

#4 Obesity:ti,ab OR obes*:ti,ab OR overweight:ti,ab OR bmi:ti,ab OR weight:ti,ab OR (body:ti,ab AND mass:ti,ab AND index:ti,ab) OR (body:ti,ab AND composition:ti,ab) OR ((waist:ti,ab OR hip:ti,ab) AND circumference:ti,ab) OR (fat:ti,ab AND mass:ti,ab)

#5 'obesity'/exp OR 'body mass'/exp OR 'body composition'/exp OR 'hip circumference'/exp OR 'waist circumference'/exp OR 'fat mass'/exp

#6 #4 OR #5

#7 meta-analysis:ti,ab OR "meta analysis":ti,ab

#8 'meta analysis'/exp

#9 #7 OR #8

#10 #3 AND #6 AND #9

SCOPUS

(TITLE-ABS (probiotic* OR synbiotic* OR prebiotic*)) AND (TITLE-ABS (obesity OR obes* OR overweight OR bmi OR weight OR (body AND mass AND index) OR (body AND composition) OR ((waist OR hip) AND circumference) OR (fat AND mass))) AND (TITLE-ABS ("meta-analysis" OR "meta analysis"))

WOS

TS=(probiotic* OR synbiotic* OR prebiotic*) AND TS=(obesity OR obes* OR overweight OR bmi OR weight OR (body AND mass AND index) OR (body AND composition) OR ((waist OR hip) AND circumference) OR (fat AND mass)) AND TS= ("meta-analysis" OR "meta analysis")
