# Supplementary material for: The effects of prebiotic, probiotic or synbiotic supplementation on overweight/obesity indicators: an umbrella review of the trials’ meta-analyses
Source: Front Endocrinol (Lausanne). 2024 Mar 20;15:1277921. doi: 10.3389/fendo.2024.1277921 (PMC10987746; doi:10.3389/fendo.2024.1277921)
Supplement: Supplementary file 2 [file DataSheet_2.pdf]

**Table S2-** Results of critical appraisal of the included meta-analysis studies by AMSTAR2																	
	1	2	3	4	5	6	7	8	9	10	11	12	13	14	15	16	Summary AMSTAR2
Rao 2009	Y	PY	Y	Y	N	Y	Y	Y	Y	N	Y	Y	N	N	Y	N	Critically low
Steenhout 2009	Y	PY	Y	N	N	N	N	PY	N	N	Y	N	N	N	N	N	Critically low
Ma et al. 2013	Y	PY	Y	Y	N	Y	PY	PY	Y	N	Y	Y	N	N	N	N	Critically low
Szajewska 2013	Y	PY	Y	Y	Y	N	Y	Y	Y	N	Y	Y	Y	Y	Y	Y	Low
Park 2015	Y	PY	Y	PY	Y	N	Y	Y	Y	Y	Y	Y	Y	Y	Y	N	Low
Sun 2015	Y	Y	Y	Y	Y	Y	Y	Y	Y	Y	Y	Y	Y	Y	Y	Y	High
Zhang 2015	Y	PY	Y	Y	Y	Y	Y	Y	Y	N	Y	Y	Y	Y	Y	Y	High
Gao 2016	Y	N	Y	Y	N	N	Y	Y	Y	Y	Y	Y	Y	Y	Y	Y	Critically low
Vahdaninia 2016	Y	Y	Y	PY	N	N	N	N	Y	N	Y	Y	Y	Y	N	N	Critically low
Dror et al. 2017	Y	N	Y	PY	N	Y	N	Y	N	N	Y	N	Y	Y	N	Y	Critically low
Lavekar 2017	Y	N	Y	PY	Y	N	PY	Y	PY	N	Y	N	N	Y	N	Y	Critically low
Sun 2017	Y	Y	Y	Y	N	N	Y	Y	Y	Y	Y	N	Y	Y	Y	Y	Low
ThompsoN 2017	Y	Y	Y	Y	Y	N	Y	Y	Y	N	Y	Y	Y	Y	Y	Y	Low
Borgeraas et al. 2018	Y	Y	N	Y	Y	N	PY	Y	Y	Y	Y	Y	Y	Y	Y	Y	Low
Han 2018	Y	Y	Y	Y	N	Y	PY	Y	Y	Y	Y	N	Y	Y	Y	Y	Moderate
John 2018	Y	Y	Y	Y	Y	Y	PY	Y	Y	Y	Y	Y	Y	Y	Y	Y	High
Loman et al. 2018	Y	PY	Y	Y	Y	Y	N	Y	Y	N	Y	Y	Y	Y	Y	Y	Moderate
Chatzakis 2019	Y	Y	Y	Y	Y	Y	Y	Y	Y	Y	Y	Y	Y	Y	Y	Y	High
Dong et al. 2019	Y	Y	Y	PY	Y	N	PY	Y	Y	N	Y	Y	Y	N	N	Y	Critically low
Hadi et al. 2019	Y	PY	N	Y	Y	Y	PY	Y	Y	N	Y	Y	Y	Y	Y	Y	Moderate
Jarde 2019	Y	Y	Y	PY	Y	Y	PY	Y	Y	Y	Y	Y	Y	Y	N	Y	Low
Koutnikova et al. 2019	Y	Y	Y	Y	Y	Y	N	Y	Y	N	Y	Y	Y	Y	Y	Y	Moderate
Liu et al. 2019	Y	PY	Y	Y	N	Y	PY	Y	Y	N	Y	Y	Y	Y	Y	Y	Moderate

Mohammadi et al. 2019	Y	PY	Y	Y	N	Y	PY	Y	Y	N	Y	Y	Y	Y	Y	Y	Moderate
Pan 2019	Y	PY	Y	Y	Y	Y	Y	Y	Y	N	Y	N	N	Y	Y	N	Low
Rahmani 2019	Y	PY	Y	Y	Y	N	N	Y	Y	Y	Y	Y	Y	Y	Y	Y	Low
Rao 2019	Y	PY	Y	Y	Y	Y	PY	Y	Y	Y	Y	Y	Y	Y	N	Y	Low
Sharpton 2019	Y	Y	Y	Y	Y	Y	Y	Y	Y	Y	Y	Y	Y	Y	Y	N	High
Snelson 2019	Y	Y	Y	Y	Y	Y	PY	Y	Y	Y	Y	Y	Y	Y	Y	Y	High
Suzumura, 2019	Y	Y	Y	Y	Y	Y	Y	Y	Y	Y	Y	Y	Y	Y	Y	Y	High
Tang 2019	Y	Y	Y	PY	Y	Y	Y	Y	Y	Y	Y	Y	Y	Y	Y	Y	High
Cao 2020	Y	Y	Y	Y	Y	Y	Y	Y	Y	Y	Y	Y	Y	Y	Y	Y	High
Wang 2019	Y	PY	Y	Y	Y	Y	Y	Y	Y	N	Y	Y	Y	Y	Y	Y	High
Xiao 2019	Y	PY	Y	Y	Y	Y	Y	Y	Y	N	Y	Y	Y	Y	Y	Y	High
Abdeloadir 2020	Y	PY	Y	Y	N	Y	PY	Y	Y	Y	Y	Y	Y	Y	Y	Y	High
Chi et al. 2020	Y	PY	N	PY	N	Y	PY	Y	Y	N	Y	Y	N	Y	N	Y	Critically low
Companys 2020	Y	Y	Y	Y	Y	N	Y	Y	Y	N	Y	Y	Y	Y	N	Y	Critically low
Cozzolin et al. 2020	Y	Y	Y	Y	Y	Y	PY	Y	Y	N	Y	Y	N	Y	N	Y	Critically low
Dixon et al. 2020	Y	Y	Y	Y	Y	N	PY	Y	Y	N	Y	Y	Y	Y	Y	Y	Low
Hadi et al. 2020	Y	PY	Y	Y	N	Y	PY	Y	Y	N	Y	Y	Y	Y	Y	Y	Moderate
Kocsis et al. 2020	Y	Y	Y	PY	Y	Y	PY	Y	Y	N	Y	Y	Y	Y	Y	Y	High
López-Moren et al. 2020	Y	Y	N	Y	Y	Y	PY	Y	Y	N	Y	Y	Y	Y	Y	Y	Moderate
Okesene-Gafa et al. 2020	Y	N	Y	Y	Y	Y	Y	Y	Y	Y	Y	Y	Y	N	Y	Y	Low
Skonieczna-żydecka 2020	Y	PY	Y	Y	Y	Y	Y	Y	Y	Y	Y	Y	Y	Y	Y	Y	High
Stachowska 2020	Y	PY	Y	PY	Y	Y	Y	Y	Y	Y	Y	Y	Y	Y	Y	Y	High
Swierz 2020	Y	Y	Y	Y	Y	Y	Y	Y	Y	Y	N	Y	Y	Y	Y	Y	Low
Wang 2020	Y	Y	Y	Y	Y	Y	Y	Y	Y	Y	Y	Y	Y	Y	Y	Y	High

Zhu 2020	Y	PY	Y	Y	Y	Y	Y	Y	Y	Y	Y	Y	Y	Y	Y	Y	High
Bock 2021	Y	Y	Y	Y	Y	Y	PY	Y	Y	Y	Y	Y	Y	Y	Y	N	High
Davidson et al. 2021	Y	Y	Y	Y	Y	Y	Y	Y	Y	Y	Y	Y	Y	Y	Y	Y	High
Hadi et al. 2021	Y	Y	Y	Y	Y	Y	Y	Y	Y	N	Y	Y	N	Y	N	Y	Critically low
Jafarabadi et al. 2021	Y	PY	Y	PY	N	N	PY	Y	N	N	Y	Y	N	N	Y	Y	Critically low
Jin et al. 2021	N	N	Y	PY	N	N	Y	Y	Y	N	Y	Y	Y	Y	Y	N	Critically low
Miao 2021	Y	Y	Y	Y	Y	Y	PY	Y	Y	Y	Y	Y	Y	Y	Y	Y	High
Mirashrafi et al. 2021	Y	Y	Y	Y	Y	Y	PY	Y	Y	N	Y	Y	Y	Y	Y	Y	High
Mohammadi et al. 2021	Y	Y	Y	Y	Y	N	PY	Y	Y	N	Y	Y	Y	Y	Y	Y	Low
Moravejolahkami et al. 2021	Y	Y	Y	Y	Y	Y	Y	Y	Y	Y	Y	Y	Y	N	Y	Y	High
Ojo 2021	Y	PY	Y	Y	Y	Y	Y	Y	Y	Y	Y	Y	Y	Y	N	Y	Low
Pan 2021	Y	PY	Y	Y	N	Y	Y	Y	Y	Y	Y	Y	Y	Y	Y	Y	High
Pérez-Castillo 2021	Y	Y	Y	Y	N	Y	Y	Y	Y	Y	Y	Y	N	Y	Y	Y	Low
Perna 2021	Y	PY	Y	PY	N	N	N	Y	Y	Y	Y	Y	Y	Y	N	Y	Critically low
Pontes 2021	Y	Y	Y	PY	N	N	Y	Y	Y	Y	Y	Y	Y	Y	Y	Y	Low
Qu 2021	Y	PY	Y	Y	Y	Y	Y	Y	Y	Y	Y	Y	Y	Y	Y	Y	High
Xu 2021	Y	Y	Y	Y	Y	Y	Y	Y	Y	Y	Y	Y	Y	Y	Y	Y	High
Yang 2021	Y	PY	Y	Y	Y	Y	N	Y	Y	Y	Y	Y	Y	Y	N	Y	Low
Zhang 2021	Y	PY	Y	Y	Y	Y	Y	Y	Y	N	Y	Y	Y	Y	Y	Y	High
Zhou 2021	Y	PY	Y	Y	N	Y	Y	Y	Y	Y	Y	Y	Y	Y	Y	Y	High
Arabi 2022	Y	Y	Y	Y	Y	Y	Y	Y	Y	Y	Y	Y	Y	Y	Y	Y	High
Chu 2022	Y	PY	Y	Y	Y	Y	Y	Y	Y	Y	Y	Y	Y	Y	Y	Y	High

Daghmouri et al. 2022	Y	Y	Y	Y	Y	Y	Y	Y	Y	N	Y	Y	Y	Y	Y	Y	High
Gkiourtzis et al. 2022	Y	Y	N	PY	Y	Y	N	PY	Y	N	Y	Y	N	Y	N	Y	Critically low
Hadi 2022	Y	PY	Y	PY	N	N	N	Y	Y	Y	Y	Y	N	Y	N	Y	Critically low
Huang et al. 2022	Y	PY	Y	PY	N	Y	PY	Y	N	N	Y	Y	Y	Y	Y	Y	Low
Kanchanasurakit et al, 2022	Y	Y	Y	Y	Y	Y	Y	Y	Y	Y	Y	Y	Y	Y	??	Y	High
Li Z et al. 2022	Y	Y	Y	Y	Y	Y	Y	Y	Y	N	Y	Y	Y	Y	Y	Y	High
Monicaasun et al. 2022	Y	Y	Y	Y	N	N	PY	PY	Y	N	Y	Y	Y	Y	N	Y	Critically low
Naseri 2022	Y	PY	Y	Y	Y	Y	Y	Y	Y	Y	Y	Y	Y	Y	Y	Y	High
Ojo 2022	Y	Y	Y	Y	Y	Y	Y	Y	Y	Y	Y	Y	Y	Y	N	Y	Low
Peckmezian 2022	Y	PY	Y	Y	Y	Y	Y	Y	Y	Y	Y	Y	Y	Y	Y	Y	High
Tabrizi 2022	Y	PY	Y	Y	Y	Y	Y	Y	Y	Y	Y	Y	Y	Y	Y	Y	High
Wang 2022	Y	PY	Y	PY	Y	Y	N	Y	Y	Y	Y	Y	Y	Y	N	Y	Low
Xing 2022	Y	PY	Y	Y	Y	Y	Y	Y	Y	Y	Y	Y	Y	Y	Y	Y	High
Zhu 2022	Y	Y	Y	PY	N	N	N	N	Y	N	Y	Y	Y	Y	N	N	Critically low
Janmohammadi et al. 2023	Y	PY	Y	Y	N	N	PY	Y	Y	N	Y	Y	Y	Y	Y	Y	Low
Li Y et al. 2023	Y	Y	Y	Y	Y	Y	PY	Y	Y	N	Y	Y	Y	Y	Y	Y	High
Mayta-Tovalin et al. 2023	Y	Y	Y	PY	Y	Y	PY	Y	Y	N	Y	Y	Y	Y	N	Y	Low
Mu et al. 2023	Y	Y	Y	PY	N	N	PY	Y	Y	N	Y	Y	Y	Y	N	Y	Critically low
Panchal 2023	Y	Y	Y	Y	N	Y	Y	Y	Y	Y	Y	Y	Y	Y	Y	Y	High
Saufani 2023	Y	PY	Y	N	N	N	Y	N	Y	N	Y	N	N	N	N	N	Critically low
Soltani 2023	Y	Y	Y	Y	Y	Y	Y	Y	Y	Y	Y	??	N	Y	Y	Y	Low
Thu 2023	Y	Y	Y	PY	Y	Y	Y	Y	Y	Y	Y	Y	Y	Y	N	Y	Low

Vazquez-Marroquin 2023	Y	Y	Y	PY	Y	Y	Y	Y	Y	Y	Y	Y	Y	Y	Y	Y	High
Wang 2023	Y	Y	Y	PY	Y	Y	Y	Y	Y	Y	Y	Y	Y	Y	Y	Y	High
Yefet 2023	Y	Y	Y	Y	Y	Y	N	Y	Y	Y	Y	Y	Y	Y	Y	Y	High
Zhou 2023	Y	PY	Y	PY	Y	Y	Y	Y	Y	Y	Y	Y	Y	Y	N	Y	High
Total percent<br>Y<br>N<br>PY	 98.95<br>1.05<br>-----	 52.63<br>5.26<br>42.11	 94.74<br>5.26<br>-----	 71.58<br>2.11<br>26.32	 70.53<br>29.47<br>-----	 74.74<br>25.26<br>-----	 54.74<br>13.68<br>31.58	 92.63<br>3.16<br>4.21	 94.74<br>4.21<br>1.05	 55.79<br>44.21<br>-----	 98.95<br>1.05<br>-----	 92.63<br>7.37<br>----	 85.26<br>14.74<br>-----	 91.58<br>8.42<br>----	 71.58<br>28.42<br>-------	 88.42<br>11.58<br>-----	

Legend: AMSTAR2: A Measurement Tool to Assess Multiple Systematic Reviews–2; **Y:** yes; **N:** No; **PY:** partial yes.
