# Supplementary material for: The effects of prebiotic, probiotic or synbiotic supplementation on overweight/obesity indicators: an umbrella review of the trials’ meta-analyses
Source: Front Endocrinol (Lausanne). 2024 Mar 20;15:1277921. doi: 10.3389/fendo.2024.1277921 (PMC10987746; doi:10.3389/fendo.2024.1277921)
Supplement: Supplementary file 3 [file Table_1.pdf]

Table 1. Characteristics of included meta−analyses of the clinical trials in general population

Reference	Design	Study Population	Total (N)	Sex (B, F, M)	Age (mean, range (y))	Included Studies (N)	Intervention/control	Intervention		Quality Assessment (yes/no)	Effects Model	MA metric (MD, WMD, SMD)	MA Outcomes			Heterogeneity		GRADE level
								dose (mg, g,CFU ,…)	duration (w)				Estimates	95% CI	p Value	I^2 (%)	p Value	
**BMI**																		
Ma et al. 2013	DBRCT	NAFLD/NASH	134	B	18-70	4	Probiotics/placebo	NR	8-24	yes	Fixed	MD	0.05	-0.18, 0.29	0.64	0	0.77	⊕◯◯◯ Very Low
Park et al. 2015	RCT	obesity	154	B	18-65	9	Probiotic/placebo, No probiotic	10^6-10^{10}	3- 24	yes	Fixed	MD	0.77	−0.24, 1.78	0.14	0	0.5	⊕⊕⊕◯ Moderate
Sun et al. 2015	RCT	CVD risk factors	788	B	24-63	15	Probiotic/placebo	10^6-5×10^{10}	4- 16	yes	Random	MD	−0.53	−0.81, −0.25	0.000	0	0.59	⊕⊕◯◯ Low
Zhang et al, 2015	RCT	Overweight, NAFLD, obese,T2DM,hypercholesterolemia,hypertension,MetS	1931	B	18–75	21	Probiotic/ control	10^6-10^{12} CFU/d	3 -24	yes	Random	MD	−0.49	−0.74, −0.24	<0.01	92	<0.1	⊕◯◯◯ Very Low
Gao et al 2016	RCT	NAFLD	535	B	5-87	4	Probiotic/placebo	NR	4-24	yes	Random	MD	-0.05	-0.36, 0.27	Non-sig.	63.3	0.043	⊕⊕⊕◯ Moderate
Lavekar et al. 2017	DBRCT	NAFLD	296	B	NR	7	Probiotic/control	NR	8-28	no	Random	WMD	−1.45	−3.06, 0.16	<0.0001	97.48	<0.0001	⊕⊕◯◯ Low

Thompson et al 2017	RCT	Overweight, obesity	656	B	18-70	12	Soluble fiber/placebo	3-34 g/d	2- 17	yes	Random	MD	-0.84	-1.35, -0.32	0.001	95	<0.00001	⊕⊕⊕◯ Moderate
Borgeraas et al. 2018	DBRCTs	Overweight, Obesity	957	B	18-75	15	Probiotics/ control	1.0 × 10^{9} - 4.8 × 10^{11} CFU	3- 12	yes	Random	WMD	-0.27	-0.45, -0.08	Sig.	56.8	0.006	⊕⊕⊕◯ Moderate
Dong et al. 2019	DBRCTs	MetS	1,544	NR	> 12	18	Probiotics/control	10^{7}-10^{11} CFU/g	3-24	no	Random	SMD	–0.34	–0.68, 0.01	0.06	74	0.0003	⊕⊕◯◯ Low
Hadi et al. 2019	RCT	NAFLD	419	B	42-57	7	Synbiotic/control	2 ×10^{7} -5×10^{9} and125 mg-10gprebiotic	8-28	yes	Random	MD	-0.08	-0.30, 0.14	0.46	12	0.339	⊕⊕◯◯ Low
John et al, 2018	DBRCT	Overweight, obese	NR	NR	NR	21	Probiotic/placebo	0.007 × 109-150 ×109	2–24	yes	Random	MD	-0.33	-0.47, -0.18	<0.0001	59	0.002	⊕⊕◯◯ Low
							Prebiotic/placebo					MD	-0.27	-0.56, 0.02	0.07	52	0.08	⊕⊕◯◯ Low
							Synbiotic/placebo					MD	-0.41	-1.48, 0.65	0.45	95	<0.00001	⊕⊕◯◯ Low
Loman et al. 2018	RCT	NAFLD	1309	B	10-77	25	Pro,pre,synbiotics/placebo	4.42 × 10^{6}-6 ×10^{10}	2-24	yes	Random	MD	-0.39	-0.49, -0.29	P<0.001	97.9	0	⊕⊕◯◯ Low
Koutnikova et al. 2019	RCT	Obesity, T2DM, NAFLD	6826	NR	NR	105	Probiotics/ control	10^{7} - 10^{12} CFU	2–28	yes	Random	MD	– 0.33	–0.53, –0.12	NR	86.3	NR	⊕⊕⊕◯ Moderate
Liu et al. 2019	RCT	NAFLD	761	B	10-58	15	Probiotics, Synbiotics / control	0.5-300 g/d	8-28	yes	Random	MD	0	-0.22, 0.22	0.99	0	0.79	⊕⊕◯◯ Low

Mohammadi et al. 2019	RCT	Overweight, obese children and adolescents	410	B	7-16	9	Pro-/synbiotics compare Placebo	2.0×10^{8}-10^{10}	4-16	yes	Random	WMD	-0.09	-0.23, 0.05	0.19	85	P< 0.001	⊕⊕⊕◯ Moderate
Rahmani et al 2019	RCT	Overweight, obese, T2DM, MetS	1065	B	31-59	20	prebiotic/placebo	0.88-9.9 mg/d	7.40 ± 7.25 Dose response	yes	Random	WMD	-0.62	-1.04, -0.21	Sig.	0	0.907	⊕⊕⊕◯ Moderate
Rao et al. 2019	RCT	T2DM	1129	B	40-65	11	Inulin-Type Carbohydrates/control	2.7 -10 g/d	6- 12	yes	Random	MD	0.07	-0.72, 0.85	0..87	0	0.55	⊕⊕⊕◯ Moderate
Sharpton et al 2019	RCT	NAFLD	1252	B	NR	21	Probiotics/control	NR	8 -28	yes	Random	WMD	-1.84	-3.30, -0.38	Sig.	23.6	0.27	⊕⊕◯◯ Low
							Synbiotics/control					WMD	-0.85	-2.17, 0.47	Non-sig.	96.6	<0.001	⊕⊕◯◯ Low
Suzumura et al., 2019	RCT	Overweight, obese	1412	B	19–65	19	Probiotic, synbiotic/control	4.97×10^{6}-1.5×10^{11}	3-24	yes	Random	MD	−0.19	−0.43, 0.04	0.11	51	0.01	⊕⊕⊕◯ Moderate
			944			15	Probiotic/control					MD	−0.18	−0.43, 0.07	0.17	58	0.005	⊕⊕⊕◯ Moderate
			301			4	Synbiotics/control					MD	−0.33	−1.22, 0.57	0.48	0	0.45	⊕⊕⊕◯ Moderate

Tang et al., 2019	RCT	NAFLD	1356	B	9-74	18	Probiotic/control	NR	4 -24	yes	Random	WMD	−1.08	−2.05, −0.11	0.03	0	89.7	⊕⊕⊕◯ Moderate
Wang et al., 2019	RCT	Healthy obese, overweight	821	B	NR	12	Probiotic/control	1×10^{7}-2×10^{11} CFU	8-24	yes	Random	MD	−0.3	−0.43, −0.18	<0.00001	59	0.006	⊕⊕⊕◯ Moderate
Xiao et al., 2019	RCT	NAFLD	1555	B	10-55	28	Probiotic/placebo	NR	4-28	yes	Random	WMD	−1.46	−2.44, −0.48	0.003	97	<0.00001	⊕⊕⊕◯ Moderate
AbdelQadir et al 2020	RCT	diabetic nephropathy	500	B	50-63	6	Probiotic soy milk vs conventional soy milk	Varied	8-12	yes	Fixed	MD	-0.07	-0.11, -0.03	0.002	73	0.05	⊕⊕⊕◯ Moderate
Cao et al. 2020	RCT	Overweight/ obese	2105	B	18–85	32	Probiotic or symbiotic/ placebo	10^{8}-1.5× 10^{11}	3-12	yes	Random	WMD	-0.25	−0.33, −0.17	NR (Sig.)	96	<0.001	⊕⊕⊕◯ Moderate
Chi et al. 2020	DBRCTs	Hypertension	846	NR	31 - 61	14	Probiotics/ control	1 × 10^{8}- 7.5 × 10^{12}	3 - 24	yes	Random	MD	-1.03	-1.28, -0.79	< 0.00001	0	0.7	⊕⊕⊕◯ Moderate
Companys et al. 2020	RCTs and observational study	obesity/overweight, T2DM, hypercholesterolemia, MetS	1,324,640	NR	18 - 75	37 RCT+ 18	Probiotic supplementation into a dairy matrix /control	1× 10^{4} - 27 × 10^{10} CFU/d	6.4-24	yes	Random	WMD	−0.33	−0.51, −0.16	<0.001	56.7	0.042	⊕⊕⊕◯ Moderate
							Probiotic with powde					WMD	−0.35	−0.48, −0.22	< 0.001	36.7	0.076	⊕⊕⊕◯ Moderate

							r or capsules/control											
Cozzolino et al. 2020	RCT	PCOS	587	F	15-48	9	Probiotics, synbiotics/ placebo	10^{8}-2 ×10^{10}	8-12	yes	Random	MD	-0.25	-0.48, -0.03	0.03	97	< 0.00001	⊕⊕◯◯ Low
Dixon et al. 2020	DBRCTs	hypertension,overweight,dyslipidemia,DM	2177	B	10-77	34	probiotics/control	3.7 × 10^{6}-7 × 10^{11} CFU	NR	yes	Random	MD	-0.31	-0.41, -0.21	<0.00001	0	0.51	⊕⊕⊕◯ Moderate
Hadi et al. 2020	RCT	Overweight, obesity	1357	B	25-77	23	Synbiotic/placebo	1×10^{7}-2×10^{11}	4 - 28	yes	Random	WMD	-0.12	-0.40, 0.16	0.39	0	1	⊕⊕◯◯ Low
Hadi et al. 2020	RCT	PCOS	540	F	25-30	8	pro-/synbiotic/control	2 × 10^{8}, 3 × 10^{10} CFU/g	8-12	yes	Random	MD	-0.23	-0.55, 0.08	0.14	92.6	NR	⊕⊕◯◯ Low
Kocsis et al. 2020	RCT	T2DM	1737	B	NR	32	Probiotics/placebo	2 × 10^{7} - 120 × 10^{9}	4-34	yes	Random	WMD	− 0.17	−0.38, 0.04	0.114	86.6	< 0.001	⊕⊕◯◯ Low
López-MoreNo et al. 2020	RCT	Overweight, Obese	329	B	NR	6	Probiotics/placebo	1 ×10^{8} ,1.35 × 10^{15} CFU/d	4-24	yes	Random	SMD	-1.71	-3.55, 0.14	0.07	98	< 0.00001	⊕◯◯◯ Very Low
Skonieczna−żydecka et al. , 2020	RCT	Healthy overweight / obese	1256	B	44.26 ± 12.87	16	Probiotic/ control	10^{6}, 10^{10} CFU/d	4-26.6	yes	Random	SMD	−0.16	−0.27, −0.05	0.006	0	0.618	⊕◯◯◯ Very Low
Stachowska et al. 2020	RCT	NAFLD	242	B	38−52	6	Prebiotic (fiber)/ control	10 - 16 g	10- 12	yes	Random	SMD	-0.49	−0.86, −0.13	0.009	41.27	0.16	⊕⊕⊕◯ Moderate
Swierz et al., 2020	RCT	morbid obesity undergoing bariatric surgery	226	B	30-44	4	Probiotics/ control	Varied	2-48	yes	Random	MD	−0.7	−2.88, 1.47	Non-sig.	84	NR	⊕◯◯◯ Very Low

Bock et al. 2021	RCT	Diabetes	2086	B	NR	38	Probiotics, prebiotics or synbiotics/control	NR	4.3-24	yes	Random	MD	−0.06	-0.53, 0.41	0.81	0	0.87	⊕⊕◯◯ Low
Hadi et al. 2021	RCT	MetS	344	B	44-63	9	Probiotic or Synbiotic/control	10^6-10^{11} CFU	3-28	yes	Random	MD	-0.06	-0.56, 0.44	0.81	0	0.989	⊕⊕⊕◯ Moderate
Jafarabadi et al. 2021	DBRCT	T2DM	NR	NR	NR	14	probiotics/control	10^7 - 1010 CFU	4 -24	no	Random	SMD	-0.02	-0.17-0.13	NR	9.9	0.35	⊕◯◯◯ Very Low
Jin et al. 2021	RCT	NAFLD	1301	B	8-70	22	Probiotic/ control	1.0 × 10^9 - 12 × 10^9 CFU	4-32	yes	Random	OR	-0.93	-1.59, 0.26	Non-sig.	NR	NR	⊕◯◯◯ Very Low
							Prebiotic/ control	10g/d-total 1.5 g				OR	-0.17	-1.28, 0.93	Non-sig.	NR	NR	⊕◯◯◯ Very Low
							synbiotic/ control	500 mg-2× 10^8 CFU				OR	-3.53	-6.47, -0.58	Sig.	NR	NR	⊕◯◯◯ Very Low
Miao et al 2021	RCT	PCOS	486	F	NR	7	Probiotic, synbiotic/control	NR	8-12	yes	Fixed	MD	-0.74	-1.58, 0.11	0.09	0	0.92	⊕⊕⊕◯ Moderate
Mirashrafi et al. 2021	RCT	Relapsing--remitting multiple sclerosis patients	213	B	34-40	4	Probiotics/control	$2×10^9$ - $4×10^9$ CFU/d	12- 24	yes	Random	WMD	0.04	-0.92, 1.00	0.939	0	0.995	⊕⊕◯◯ Low
Mohammadi et al. 2021	DBRCT	Overweight, obesity, hypertensive, DM	1519	B	35-80	19	Probiotics fermented	4.97 × 10^6-7 × 10^{11}	2-12	yes	Random	WMD	-0.33	-0.48, -0.18	< 0.001	0	0.994	⊕⊕⊕◯ Moderate

							milk products /placebo											
Ojo et al. 2021	RCT	T2DM	3295	B	40-75	10	Dietary Fibre/control or placebo	NR	3- 52	yes	Fixed	MD	−0.57	−1.02, −0.12	0.01	0	0.51	⊕⊕⊕⊕ High
Pan et al. 2021	RCT	MetS	5708	B	13-72	42	Microbial therapy/control	Varied	Once dose-52 w	yes	Random	SMD	−0.13	−0.27, 0.00	0.05	0	0.86	⊕⊕⊕◯ Moderate
Perna et al. 2021	RCT	Overweight, Obese	1411	B	10-75	20	Probiotic/placebo	10^6- 1.5 × 10^{11}	3-24	yes	Random	MD	−0.73	−1.31, −0.16	0.01	100	<0.00001	⊕⊕◯◯ Low
Pontes et al. 2021	RCT	Overweight, obese	1720	B	31-66	26	Probiotic/placebo	2×10^7 - 112.5 × 10^9 CFU/d	3-24	yes	Random	MD	−0.24	−0.35, −0.12	0.0001	55	0.003	⊕⊕◯◯ Low
Qu et al. 2021	RCT	Overweight, Obese	535	B	10-70	12	Prebiotic/ placebo	2-21 g/d	2- 25.7	yes	Random	WMD	−0.2	−0.58, 0.19	0.32	>50	<0.001	⊕⊕◯◯ Low
Xu et al 2021	RCT	T2D	1995	B	35-69	45	High, low Microbiota−accessibleCH	Varied	6 -12	yes	Random	WMD	−0.48	−0.64, −0.31	Sig.	0	0.681	⊕⊕⊕⊕ High
Yang et al, 2021	DBRCT	NAFLD	352	B	≥18	6	Probiotic / control	Varied	8-48	yes	Random	WMD	−0.68	−1.97, 0.6	0.3	99.3	<0.00001	⊕◯◯◯ Very Low
Zhang et al, 2021	RCT	Morbid Obesity after	172	NR	NR	4	Probiotic/ control	1 × 10^8- 1 × 10^{10} CFU/g	2-24	yes	Random	12 w after surgery, MD	0.01	−0.84, 0.85	0.99	77	0.01	⊕◯◯◯ Very Low

		Bariatric Surgery									Fixed	48w after surgery, MD	−0.25	−1.47, 0.96	0.68	0	0.4	⊕◯◯◯ Very Low
Arabi et al. 2022	DBRCT	MetS	1049	B	40-72	5	Synbiotic/placebo	2×10^8 CFU - 6×10^9 CFU/d	8-28	yes	Random	WMD	−0.78	−1.56, 0.01	0.05	91.4	<0.001	⊕◯◯◯ Very Low
Daghmouri et al. 2022	RCT	Morbid obesity under bariatric surgery	279	NR	34-45	5	Probiotics/placebo	1×10^8 -1 $\times 10^{10}$ CFU/g	12-24	yes	Random	MD	0.63	-1.05, 2.31	0.46	97	< 0.01	⊕◯◯◯ Very Low
Gkiourtzis et al. 2022	RCT	NAFLD children	238	B	10-13	5	Probiotics/placebo	NR	8-16	yes	Random	MD	-2	-3.07, -0.93	<0.0001	36	0.21	⊕⊕⊕◯ Moderate
Huang et al. 2022	RCT	NAFLD	1403	NR	NR	24	probiotics/placebo	NR	4-24	yes	Random	MD	−0.80	−1.51, −0.08	0.03	89	< 0.0001	⊕⊕◯◯ Low
Kanchanasurakit et al, 2022	Network, RCT	NAFLD	1,230	B	18-77	13	Prebiotic/control	Varied	4-48	yes	Random	WMD	−0.36	−1.69, 0.97	Non-sig.	NR	NR	⊕⊕⊕⊕ High
							Probiotic/ control						−0.25	−1.47, 0.97				⊕⊕⊕⊕ High
							Synbiotic/ control						−0.71	−1.49, 0.06				⊕⊕⊕⊕ High
Li Z et al. 2022	RCT	PostmeNopausal with Overweight, obese	281	F	53-62	6	Probiotics/control	2.5×10^9 ,9.4×10^{10} CFU/d	5-12	yes	Random	MD	-0.34	-0.72, 0.03	0.07	0	0.94	⊕⊕⊕◯ Moderate
Naseri et al. 2022	DBRCT	Prediabetes, T2DM	3537	B	35–66	54	Synbiotic or probiotics/control	3.7×10^6 - 1×10^{11} CFU /g	6-24	yes	Random	MD	−0.06	−0.18 ,0.05	0.202	0	0.977	⊕⊕⊕◯ Moderate

Ojo et al. 2022	RCT	T2DM	1078	B	48.5-67	14	Prebiotics and oral anti−diabetic agents/control or placebo	NR	1.3- 52	yes	Fixed	SMD	-0.22	-0.46, 0.02	0.07	33	0.2	⊕⊕⊕⊕ High
Peckmezian et al. 2022	RCT	Overweight with/without major health condition	309	B	41.7 ± 8.9	11	Probiotic/control	1.79 x 10^{6}-2 x 10^{10}	3-28	yes	Random	MD	−0.22	−0.45, 0.02	Non-sig.	0	0.5	⊕⊕⊕◯ Moderate
			231			10	Synbiotic/control					MD	−0.05	−0.38, 0.28	Non-sig.	0	0.98	⊕⊕⊕◯ Moderate
Tabrizi et al., 2022	DBRCT	PCOS	730	F	24-31	11	Probioti/placebo	2×10^{8}-3×10^{10} CFU/g	8-12	yes	Random	SMD	−0.29	−0.54, −0.03	0.02	50.7	0.039	⊕⊕⊕⊕ High
Zhu et al., 2022	RCT	Obese	1818	B	47.9	32	Probiotic/placebo	NR	NR	NR	Random	MD	-0.36	-0.44, -0.29	<0.01	NR	NR	⊕⊕⊕⊕ High
Wang et al. 2022	RCT	NAFLD	1,136	B	9-74	18	Probiotic/control	NR	4- 48	yes	Random	MD	−1.67	−2.93, −0.41	0.009	95	<0.00001	⊕⊕⊕⊕ High
Xing et al., 2022	RCT	NAFLD, healthy	741	B	11-54	11	Probiotics, prebiotics, symbiotics/control	Varied	8 -96	yes	Fixed	SMD	−0.01	−0.17, 0.15	Non-sig.	0	0.995	⊕⊕⊕⊕ High
Li Y et al. 2023	RCT	PCOS	1049	F	NR	17	Probiotics, prebiotics, synbiotics/	10^{7} - 10^{10} CFU	8-12	yes	Random	SMD	-0.1	-0.3, 0.09	0.295	47.3	0.03	⊕⊕⊕⊕ High

							placebo											
Mayta-TovaliNo et al. 2023	RCT	Overweight, obese	2170	B	28 - 68	25	Probiotics/placebo	1.79 $\times 10^{6}$- 1.8 $\times 10^{9}$	8-24	yes	Random	MD	-0.27	-0.35, -0.19	NR	26	0.16	⊕⊕⊕⊕ High
Saufani et al. 2023	RCT	MetS	630	B	18-65	11	Probiotic/placebo	10^{6} - 10^{9} CFU/d	4- 30	yes	Random	MD	−0.55	−0.82, −0.27	Sig.	NR	NR	⊕⊕⊕⊕ High
Soltani et al., 2023	RCT	Diabetes	1387	B	44-65	27	Probiotics/synbiotics/control	1 × 10^{8} - 3 × 10^{11} CFU/d	4- 26	yes	Random	WMD	−0.2	−0.33, −0.07	0.003	78.4	<0.001	⊕⊕◯◯ Low
Thu et al., 2023	RCT	Breast cancer	571	F	18-75	13	Probiotics/control	Varied	2–10	yes	Random	MD	−0.32	−1.01, 0.38	0.37	0	0.45	⊕⊕⊕⊕ High
Wang et al., 2023	RCT	NAFLD	266	B	18-75	6	Probiotic,symbiotic,prebiotic / control	NR	8-24	yes	Fixed	MD	−0.57	−0.72, −0.42	<0.00001	0	0.82	⊕⊕⊕⊕ High
Zhou et al, 2023	RCT	NAFLD	1037	B	10-64	21	Probiotic/control	NR	8-56	yes	Random	MD	−0.91	−1.99, 0.16	0.09	94	<0.00001	⊕⊕⊕⊕ High
**Weight**																		
Park et al. 2015	RCT	obesity	196	B	18-65	9	Probiotic/placebo or No probiotic	10^{6}-10^{10}	3- 24	yes	Fixed	MD	−1.77	−4.84, 1.29	0.26	0	0.58	⊕⊕⊕◯ Moderate

Zhang et al, 2015	RCT	Overweight, NAFLD, obese, T2DM, hypercholesterolemia, hypertension, MetS	1931	B	18–75	21	Probiotic/ control	10^6 - 10^{12} CFU/d	3 -24	yes	Random	MD	−0.59	−0.87, −0.3	<0.01	30	<0.1	⊕◯◯◯ Very Low
Dror et al. 2017	RCT	Adults	NR	NR	19 – 65	13	Pro, pre, synbiotics vs. placebo/ No treatment	varied	2-26	yes	Random	SMD	-0.43	-0.67, -0.20	0.0003	58	0.005	⊕⊕◯◯ Low
		Children	NR	NR	2w –5 yrs	17			8-104				0.20	0.04, 0.36	0.01	0	0.72	⊕⊕◯◯ Low
		Infants	NR	NR	1d-36 mo	23			3-52				0.30	-0.01, 0.62	0.05	86	<0.00001	⊕⊕◯◯ Low
Thompson et al 2017	RCT	Overweight, obesity	659	B	18-70	12	Soluble fiber/placebo	3-34 g/d	2- 17	yes	Random	MD	-2.52	-4.25, -0.79	0.004	96	<0.00001	⊕⊕⊕◯ Moderate
Borgeraas et al. 2018	DBRCTs	Overweight, Obesity	957	B	18-75	15	Probiotics/ control	1.0 × 10^9 - 4.8 × 10^{11} CFU	3- 12	yes	Random	WMD	-0.6	-1.19, -0.01	Sig.	49.1	0.023	⊕⊕⊕◯ Moderate
John et al, 2018	DBRCT	Overweight, obese	NR	NR	NR	21	Probiotic/placebo	0.007 × 109-150 ×109	2–24	yes	Random	MD	-0.65	-1.12, -0.18	0.007	71	<0.0001	⊕⊕◯◯ Low
							Prebiotic/placebo					MD	-0.90	-1.77, -0.02	0.04	86	<0.0001	⊕⊕◯◯ Low
							Synbiotic/placebo					MD	-1.09	-3.45, 1.26	0.36	96	<0.00001	⊕⊕◯◯ Low

Hadi et al. 2019	RCT	NAFLD	419	B	42-57	7	Synbiotic/control	2×10^7 - 5×10^9 and 125 mg-10g prebiotic	8-28	yes	Random	MD	- 2.98	-3.78, -2.19	<0.001	0	0.446	⊕⊕◯◯ Low
Koutnikova et al. 2019	RCT	Obesity, T2DM, NAFLD	6826	NR	NR	105	Probiotics/ control	10^7 - 10^{12} CFU	2–28	yes	Random	MD	-0.39	−0.57, −0.21	NR	22.6	NR	⊕⊕⊕◯ Moderate
Mohammadi et al. 2019	RCT	Overweight, obese children and adolescents	410	B	7-16	9	Pro-/synbiotics Vs.Placebo	2.0×10^8-10^{10}	4-16	yes	Random	WMD	0.70	-1.07, 2.47	0.43	0	0.97	⊕⊕⊕◯ Moderate
Rahmani et al 2019	RCT	Overweight, obese, T2DM, MetS	1065	B	31-59	20	prebiotic/placebo	0.88-9.9 mg/d	7.40 ± 7.25 Dose response	yes	Random	WMD	-0.77	-1.49, -0.04	Sig.	40	0.082	⊕⊕⊕◯ Moderate
Snelson et al, 2019	RCT	Healthy, overweight /obese, MetS, T2DM	670	B	23-70	20	prebiotic/placebo	8 - 66 gr/d	1-12	yes	Random	MD	-1.19	-2.27, -0.12	0.03	0	0.99	⊕⊕⊕◯ Moderate
Suzumura et al., 2019	RCT	Overweight, obese	1412	B	19–65	19	Probiotic, synbiotic/control	4.97×10^6-1.5×10^{11}	3-24	yes	Random	MD	−0.54	−1.09, 0.01	0.05	0	0.92	⊕⊕⊕◯ Moderate
			944			15	Probiotic/control					MD	−0.42	−1.01, 0.16	0.16	0	0.86	⊕⊕⊕◯ Moderate
			301			4	synbiotic/control					MD	−1.24	−2.57, 0.1	0.07	0	0.94	⊕⊕⊕◯ Moderate

Tang et al., 2019	RCT	NAFLD	1356	B	9-74	18	Probiotic/control	NR	4 -24	yes	Fixed	WMD	−2.31	−4.45, −0.16	0.035	0	0.559	⊕⊕⊕◯ Moderate
Wang et al., 2019	RCT	Healthy obese, overweight	821	B	NR	12	Probiotic/control	1×10^{7}-2×10^{11} CFU	8-24	yes	Random	MD	−0.55	−0.91, −0.19	0.003	64	0.003	⊕⊕⊕◯ Moderate
AbdelQadir et al 2020	RCT	diabetic nephropathy	500	B	53-63	6	Probiotic/placebo	Varied	8-12	yes	Fixed	MD	0.05	-0.23, 0.33	0.73	29	0.24	⊕⊕⊕◯ Moderate
							Probiotic soy milk/ conventional soy milk					MD	-0.04	-0.19, 0.10	0.57	0	0.94	⊕⊕⊕◯ Moderate
Companys et al. 2020	RCTs and observational study	obesity/overweight, T2DM, hypercholesterolemia, MetS	1,324,640	NR	18 - 75	37 RCT+ 18	Probiotic supplementation into a dairy matrix /control	1×10^{4} - 27×10^{10} CFU/d	6.4-24	yes	Random	WMD	−0.14	−0.3, 0.02	<0.001	75.4	<0.001	⊕⊕⊕◯ Moderate
							Probiotic with powder or capsules/control					WMD	−0.26	−0.43, −0.09	0.002	66.4	0.002	⊕⊕⊕◯ Moderate
Cozzolino et al. 2020	RCT	PCOS	587	F	15-48	9	Probiotics, synbiotics/ placebo	10^{8}-2×10^{10}	8-12	yes	Random	MD	-0.75	-1.45, -0.05	0.04	97	< 0.00001	⊕⊕◯◯ Low

Hadi et al. 2020	RCT	Overweight, obesity	1357	B	25 - 77	23	Synbiotic/placebo	1×10^{7}-2×10^{11}	4 - 28	yes	Random	WMD	-0.80	-1.56, -0.03	0.04	0	0.999	⊕⊕◯◯ Low
Hadi et al. 2020	RCT	PCOS	540	F	25-30	8	pro-/synbiotic/control	2 × 108, 3 × 1010 CFU/g	8-12	yes	Random	MD	-0.67	-1.43, 0.10	0.08	90.8	NR	⊕⊕◯◯ Low
Hadi et al. 2021	RCT	MetS	344	B	44-63	9	Probiotic or Synbiotic/control	10^{6}-10^{11} CFU	3-28	yes	Random	MD	-1.54	-4.83, 1.75	0.38	0	1	⊕⊕⊕◯ Moderate
Jin et al. 2021	RCT	NAFLD	1301	B	8-70	22	Probiotic/ control	1.0 × 10^{9} - 12 × 10^{9} CFU	4-32	yes	Random	OR	-4.93	-9.72, -0.15	Sig.	NR	NR	⊕◯◯◯ Very Low
							Prebiotic/ control	10g/d-total 1.5 g				OR	-2.31	-7.65, 3.04	Non-sig.	NR	NR	⊕◯◯◯ Very Low
							Synbiotic/control	500 mg-2× 10^{8} CFU				OR	0.44	0.17, 0.72	Sig.	NR	NR	⊕◯◯◯ Very Low
Mirashrafi et al. 2021	RCT	Relapsing--remitting multiple sclerosis patients	213	B	34-40	4	Probiotics/control	2×10^{9} - 4× 10^{9} CFU/d	12- 24	yes	Random	WMD	0.16	-3.01, 3.33	0.923	0	0.998	⊕⊕◯◯ Low
Mohammadi et al. 2021	DBRCT	Overweight, obesity, hypertensive, DM	1519	B	35-80	19	Probiotics fermented milk products /placebo	4.97 × 10^{6}-7 × 10^{11}	2-12	yes	Random	WMD	-0.91	-1.59, -0.22	0.010	67.3	< 0.001	⊕⊕⊕◯ Moderate

Moravejolahkami et al. 2021	RCT	Diabetic nephropathy	340	B	50-60	6	Probiotics/control	2.5×10^9 - 8×10^9 CFU/d	8-12	yes	Random	WMD	0.22	−2.28, 2.72	0.864	0	0.999	⊕⊕◯◯ Low
Perna et al. 2021	RCT	Overweight, Obese	1411	B	10-75	20	Probiotic/placebo	10^6- 1.5×10^{11}	3-24	yes	Random	MD	−0.26	−0.75, 0.23	0.3	97	<0.00001	⊕⊕◯◯ Low
Pontes et al. 2021	RCT	Overweight, obese	1720	B	31-66	26	Probiotic/placebo	2×10^7 - 112.5×10^9 CFU/d	3-24	yes	Random	MD	−0.7	−1.04, −0.35	<0.0001	70	<0.00001	⊕⊕◯◯ Low
Qu et al. 2021	RCT	Overweight, Obese	535	B	10-70	12	Prebiotic/placebo	2-21 g/d	2- 25.7	yes	Random	WMD	−0.51	-1.18, 0.16	0.14	>50	<0.001	⊕⊕◯◯ Low
Xu et al 2021	RCT	T2D	1995	B	35-69	45	High, low Microbiota−accessibleCH	Varied	6 -12	yes	Random	WMD	−1.31	−1.71, −0.91	Sig	0	0.294	⊕⊕⊕⊕ High
Zhang et al, 2021	RCT	Morbid Obesity after Bariatric Surgery	172	NR	NR	4	Probiotic/control	1×10^8 - 1×10^{10} CFU/g	2-24	yes	Random	12w after surgery, MD	−1.82	−7.87, 4.24	0.56	87	0.006	⊕◯◯◯ Very Low
Arabi et al. 2022	DBRCT	MetS	1049	B	40-72	5	Synbiotic/placebo	2×10^8 CFU - 6×10^9 CFU /d	8-28	yes	Random	WMD	−4.3	−6.2 ,−2.5	0.001	0	0.4	⊕◯◯◯ Very Low
Daghmouri et al. 2022	RCT	Morbid obesity under bariatric surgery	279	NR	34-45	5	Probiotics/placebo	1×10^8 -1×10^{10} CFU/g	12-24	yes	Random	MD	2.71	-2.67, 8.09	0.32	98	< 0.01	⊕◯◯◯ Very Low
Naseri et al. 2022	DBRCT	Prediabetes, T2DM	3537	B	35−66	54	Synbiotic or probiotics/control	3.7×10^6- 1×10^{11} CFU /g	6-24	yes	Random	WMD	−0.38	−0.63 ,−0.12	0.004	0	<0.0001	⊕⊕⊕◯ Moderate

Peckmezian et al. 2022	RCT	Overweight with/without major health condition	309	B	41.7 ± 8.9	11	Probiotic/control	1.79 x 10^6-2 x 10^{10}	3- 28	yes	Random	MD	−0.73	−1.02, −0.44	< 0.001	14	0.31	⊕⊕⊕◯ Moderate
			231			10	Synbiotic/control					MD	−0.81	−1.83, 0.22	Non-sig.	0	1	⊕⊕⊕◯ Moderate
Tabrizi et al., 2022	DBRCT	PCOS	730	F	24-31	11	Probioti/placebo	2×10^8-3×10^{10} CFU/g	8-12	yes	Random	SMD	−0.3	−0.53, −0.07	0.01	42.5	0.084	⊕⊕⊕⊕ High
Li Y et al. 2023	RCT	PCOS	1049	F	NR	17	Probiotics, prebiotics, synbiotics/placebo	10^7 - 10^{10} CFU	8-12	yes	Random	SMD	-0.11	-0.34, 0.13	0.379	61.5	0.003	⊕⊕⊕◯ Moderate
Mayta-TovaliNo et al. 2023	RCT	Overweight, obese	2170	B	28 - 68	25	Probiotics/placebo	1.79 ×10^6-1.8 ×10^9	8-24	yes	Random	MD	-0.61	-0.89, -0.34	NR	0	0.79	⊕⊕⊕⊕ High
Soltani et al., 2023	RCT	Diabetes	1387	B	44-65	27	Probiotics/synbiotics/control	1 × 10^8 - 3 × 10^{11} CFU/d	4- 26	yes	Random	WMD	−0.62	−0.97, −0.28	<0.001	81.1	<0.001	⊕⊕◯◯ Low
Thu et al., 2023	RCT	Breast cancer	571	F	18-75	13	Probiotics/control	Varied	2–10	yes	Random	MD	0.19	−3.65, 4.03	0.92	67	0.03	⊕⊕⊕◯ Moderate
**EWL, %EWL**																		
Swierz et al., 2020	RCT	morbid obesity undergoing bariatric surgery	226	B	32-44	4	Probiotics/ control	Varied	2-48	yes	Random	%EWL, 6w, , MD	0.28	−9.53, 10.09	Non-sig.	71	NR	⊕◯◯◯ Very Low
												%EWL, 12w, MD	5.47	−3.22, 14.17	Non-sig.	82	NR	⊕◯◯◯ Very Low

												%EWL 24w, MD	0.46	−8.14, 9.07	Non-sig.	50	NR	⊕◯◯◯ Very Low
												%EWL 48w, MD	0.35	−8.66, 9.37	Non-sig.	59	NR	⊕◯◯◯ Very Low
Zhu et al, 2020	RCT	Obese patients after bariatric surgery	269	B	32-53	6	Probiotics, prebiotics, synbiotics/control	1×10^8-3.5×10^9	2 weeks and 13 months	yes	Random	EWL, SMD	0.45	−0.16, 1.05	0.15	68	0.02	⊕◯◯◯ Very Low
Zhang et al, 2021	RCT	Morbid Obesity after Bariatric Surgery	172	NR	NR	4	Probiotic/ control	1×10^8 - 1×10^{10} CFU/g	2-24	yes	Random	%EWL,12w, MD	4.48	−2.18, 11.14	0.19	73	0.01	⊕◯◯◯ Very Low
												%EWL 48w, MD	0.43	−8.78, 9.64	0.93	60	0.11	⊕◯◯◯ Very Low
**WC**																		
Sun et al. 2015	RCT	CVD risk factors	788	B	24-63	15	Probiotic/placebo	10^6-5×10^{10}	4- 16	yes	Random	MD	−2.11	−3.54, −0.68	0.004	0	0.63	⊕⊕◯◯ Low
Thompson et al 2017	RCT	Overweight, obesity	659	B	18-70	12	Soluble fiber/placebo	3-34 g/d	2- 17	yes	Random	MD	-2.01	-5.35, 1.35	0.24	97	<0.00001	⊕⊕⊕◯ Moderate
Dong et al. 2019	DBRCTs	MetS	1,544	NR	> 12	18	Probiotics/control	10^7-10^{11} CFU/g	3-24	no	Random	SMD	–0.35	–0.81, 0.11	0.14	81	0.0001	⊕⊕◯◯ Low
Hadi et al. 2019	RCT	NAFLD	419	B	42-57	7	Synbiotic/control	2×10^7 - 5×10^9 and 125 mg-	8-28	yes	Random	MD	-0.96	-2.63, 0.70	0.25	73.7	0.010	⊕⊕◯◯ Low

								10g prebiotic										
Koutnikova et al. 2019	RCT	Obesity, T2DM, NAFLD	6826	NR	NR	105	Probiotics/ control	10^7 - 10^{12} CFU	2–28	yes	Random	MD	−1.01	−1.55 ,−0.48	NR	35.6	NR	⊕⊕⊕◯ Moderate
Liu et al. 2019	RCT	NAFLD	761	B	10-58	15	Probiotics, Synbiotics / control	0.5-300 g/d	8-28	yes	Random	MD	-0.01	-0.03, 0.02	0.57	0	0.76	⊕⊕◯◯ Low
Mohammadi et al. 2019	RCT	Overweight, obese children and adolescents	410	B	7-16	9	Pro-/synbiotics compare Placebo	2.0× 10^8-10^{10}	4-16	yes	Random	WMD	-0.62	1.73, 0.48	0.26	10.7	0.34	⊕⊕⊕◯ Moderate
Rahmani et al 2019	RCT	Overweight, obese, T2DM, MetS	1065	B	31-59	20	prebiotic/placebo	0.88-9.9 mg/d	7.40 ± 7.25 Dose response	yes	Random	WMD	-0.2	-0.59, 0.18	Non-sig.	0	0.960	⊕⊕⊕◯ Moderate
Suzumura et al., 2019	RCT	Overweight, obese	1412	B	19-65	19	Probiotic, synbiotic/control	4.97×10^6-1.5×10^{11}	3-24	yes	Random	MD	−0.82	−1.43, −0.21	0.009	46	0.04	⊕⊕⊕◯ Moderate
			944			15	Probiotic/control					MD	−0.57	−1.0, −0.15	0.008	0	0.53	⊕⊕⊕◯ Moderate
			301			4	Synbiotic/control					MD	−3.39	−5.07, −1.72	<0.0001	34	0.22	⊕⊕⊕◯ Moderate
Wang et al., 2019	RCT	Healthy obese, overweight	821	B	NR	12	Probiotic/control	1×10^7-2×10^{11} CFU	8-24	yes	Random	MD	−1.20	−2.21, −0.19	0.02	90	<0.00001	⊕⊕⊕◯ Moderate

Cao et al. 2020	RCT	Overweight/ obese	2105	B	18–85	32	Probiotic or symbiotic/ placebo	10^{8}-1.5× 10^{11}	3-12	yes	Random	WMD	-0.99	−1.33, −0.66	NR (Sig.)	92.8	<0.001	⊕⊕⊕◯ Moderate
Companys et al. 2020	RCTs and observational study	obesity/overweight, T2DM, hypercholesterolemia, MetS	1,324,640	NR	18 - 75	37 RCT+ 18	Probiotic supplementation into a dairy matrix /control	1× 10^{4} - 27 × 10^{10} CFU/d	6.4-24	yes	Random	WMD	−0.49	−0.68, −0.29	<0.001	80.5	<0.001	⊕⊕⊕◯ Moderate
							Probiotic with powder or capsules/control					WMD	−0.37	−0.52, −0.21	< 0.001	53.0	0.015	⊕⊕⊕◯ Moderate
Hadi et al. 2020	RCT	Overweight, obesity	1357	B	25 - 77	23	Synbiotic/placebo	1×10^{7}-2×10^{11}	4 - 28	yes	Random	WMD	-2.07	-3.11 ,-1.03	<0.001	0	0.952	⊕⊕◯◯ Low
Skonieczna−żydecka et al. , 2020	RCT	Healthy overweight / obese	1256	B	44.26 ± 12.87	16	Probiotic/ control	10^{6} and 10^{10} CFU/d	4-26.6	yes	Random	SMD	−0.15	−0.30, 0.003	0.055	0	0.636	⊕◯◯◯ Very Low
Hadi et al. 2021	RCT	MetS	344	B	44-63	9	Probiotic or Synbiotic/control	10^{6}-10^{11} CFU	3-28	yes	Random	MD	-1.33	-3.30, 0.64	0.18	28.9	0.219	⊕⊕⊕◯ Moderate
Miao et al 2021	RCT	PCOS	486	F	NR	7	Probiotic, synbiotic/control	NR	8-12	yes	Random	MD	-1.88	-4.88, 1.12	0.22	63	0.07	⊕⊕⊕◯ Moderate

Mohammadi et al. 2021	DBRCT	Overweight, obesity, hypertensive, DM	1519	B	35-80	19	Probiotics fermented milk products /placebo	4.97 × 10^6-7 × 10^{11}	2-12	yes	Random	WMD	-0.59	-1.55, 0.36	0.221	0	0.913	⊕⊕⊕◯ Moderate
Pan et al. 2021	RCT	MetS	5708	B	12-72	42	Microbial therapy/control	Varied	Once dose-52 w	yes	Random	SMD	−0.26	−0.49, −0.03	0.03	57	0.007	⊕⊕⊕◯ Moderate
Perna et al. 2021	RCT	Overweight, Obese	1411	B	10-75	20	Probiotic/placebo	10^6-1.5 × 10^{11}	3-24	yes	Random	MD	−0.71	−1.24, −0.19	0.008	96	<0.00001	⊕⊕◯◯ Low
Pontes et al. 2021	RCT	Overweight, obese	1720	B	31-66	26	Probiotic/placebo	2×10^7 - 112.5 × 10^9 CFU/d	3-24	yes	Random	MD	−1.13	−1.54, −0.73	<0.00001	33	0.12	⊕⊕◯◯ Low
Zhang et al, 2021	RCT	Morbid Obesity after Bariatric Surgery	172	NR	NR	4	Probiotic/ control	1 × 10^8 - 1 × 10^{10} CFU/g	2-24	yes	Random	12w after surgery, MD	−2.2	−7.23, 2.83	0.39	67	0.08	⊕◯◯◯ Very Low
											Fixed	48w after surgery, MD	−4.21	−7.26, −1.15	0.007	0	0.39	⊕◯◯◯ Very Low
Arabi et al. 2022	DBRCT	MetS	1049	B	40-72	5	Synbiotic/placebo	2 × 10^8 CFU - 6 × 10^9 CFU /d	8-28	yes	Random	WMD	−4.04	−4.99 , −3.08	< 0.001	22.7	0.274	⊕◯◯◯ Very Low
Daghmouri et al. 2022	RCT	Morbid obesity under bariatric surgery	279	NR	34-45	5	Probiotics/placebo	1 × 10^8 -1 × 10^{10} CFU/g	12-24	yes	Random	MD	0.20	-7.12, 7.52	0.96	99	< 0.01	⊕◯◯◯ Very Low

Gkiourtzis et al. 2022	RCT	NAFLD children	238	B	10-13	5	Probiotics/placebo	NR	8-16	yes	Random	MD	-1.37	-2.39, -0.35	<0.008	0	0.76	⊕⊕⊕◯ Moderate
Kanchanasurakit et al, 2022	Network, RCT	NAFLD	1,230	B	18-77	8	Prebiotic/control	Varied	4-48	yes	Random	WMD	−0.79	−2.6, 1.03	Non-sig.	NR	NR	⊕⊕⊕⊕ High
							Probiotics/ control						−0.28	−2.01, 1.45	Sig.			⊕⊕⊕⊕ High
							Synbiotic/ control						−2.26	−2.98, −1.54	Sig.			⊕⊕⊕⊕ High
Naseri et al. 2022	DBRCT	Prediabetes, T2DM	3537	B	35−66	54	Synbiotic or probiotics/control	3.7×10^6- 1×10^{11} CFU /g	6-24	yes	Random	MD	− 0.61	−2.16, 0.94	0.44	0	0.961	⊕⊕⊕◯ Moderate
Peckmezian et al. 2022	RCT	Overweight with/without major health condition	309	B	41.7 ± 8.9	11	Probiotic/control	1.79 x 10^6-2 x 10^{10}	3- 28	yes	Random	MD	−0.53	−1.00, −0.07	0.024	0	0.67	⊕⊕⊕◯ Moderate
			231			10	Synbiotic/control					MD	−1.31	−2.05, −0.57	<0.001	0	0.48	⊕⊕⊕◯ Moderate
Li Y et al. 2023	RCT	PCOS	1049	F	NR	17	Probiotics, prebiotics, synbiotics/ placebo	10^7 - 10^{10} CFU	8-12	yes	Random	SMD	0.37	-0.78, 1.53	0.525	95.5	0	⊕⊕⊕◯ Moderate
Soltani et al., 2023	RCT	Diabetes	1387	B	44-65	27	Probiotics/synbiotics/ control	1×10^8 - 3×10^{11} CFU/d	4- 26	yes	Random	WMD	-0.93	-1.34, -0.53	< 0.001	0	0.974	⊕⊕⊕⊕ High

Thu et al., 2023	RCT	Breast cancer	571	F	18-75	13	Probiotics/control	Varied	2–10	yes	Random	MD	0.69	−2.98, 4.35	0.71	19	0.29	⊕⊕⊕⊕ High
Vazquez–Marroquin et al. 2023	DBRCT	T2DM	830	B	30-65	19	Probiotics, prebiotics, synbiotics/control	Probiotics: 10^8 - 20 x 10^{10} CFU/d Prebiotics: 0.1 g/d and 30 g/d	6-24	yes	Random	MD	−0.969	−1.74, −0.20	0.014	68	<0.001	⊕⊕⊕⊕ High
						11	Probiotic/control					MD	−0.62	−1.0, −0.24	0.002	0	0.925	⊕⊕⊕⊕ High
						7	Prebiotic/control					MD	−1.69	−3.74, 0.37	0.108	87	<0.001	⊕⊕⊕◯ Moderate
						1	Synbiotic/control					MD	−3.00	−7.11, 1.11	0.153	NR	NR	⊕⊕⊕⊕ High
**HC, WHR**																		
Dong et al. 2019	DBRCTs	MetS	1,544	NR	> 12	18	Probiotics/control	10^7-10^{11} CFU/g	3-24	no	Random	HC, SMD	–0.35	–0.74, 0.05	0.08	78	0.001	⊕⊕◯◯ Low
												WHR, SMD	–0.10	–0.47, 0.28	0.62	57	0.06	⊕⊕◯◯ Low
Cao et al. 2020	RCT	Overweight/ obese	2105	B	18–85	32	Probiotic or symbiotic/placebo	10^8-1.5× 10^{11}	3-12	yes	Fixed	WHR, WMD	−0.01	−0.02, 0.01	NR (Non-sig.)	15	0.314	⊕⊕⊕◯ Moderate
Moravejolahkami et al. 2021	RCT	Diabetic nephropathy	340	B	50-60	6	Probiotics/control	2.5 × 10^9 - 8 × 10^9 CFU/d	8-12	yes	Random	WHR, WMD	0.10	-0.10, 0.30	NR	0	1	⊕⊕◯◯ Low

Pontes et al. 2021	RCT	Overweight, obese	1720	B	31-66	26	Probiotic/placebo	2×10^{7} - 112.5 × 10^{9} CFU/d	3-24	yes	Random	WHR, MD	-0.01	-0.01, -0.01	<0.00001	24	0.26	⊕⊕◯◯ Low
Soltani et al., 2023	RCT	Diabetes	1387	B	44-65	27	Probiotics/synbiotics/ control	1 × 10^{8} - 3 × 10^{11} CFU/d	4- 26	yes	Random	WHR, WMD	-0.02	-0.06, 0.02	0.32	70.2	0.009	⊕⊕◯◯ Low
**BFM, BF%**																		
Thompson et al 2017	RCT	Overweight, obesity	659	B	18-70	12	Soluble fiber/placebo	3-34 g/d	2- 17	yes	Random	BFM, MD	-0.41	-0.58, -0.24	<0.00001	89	<0.00001	⊕⊕⊕◯ Moderate
Borgeraas et al. 2018	DBRCTs	Overweight, Obesity	957	B	18-75	15	Probiotics/ control	1.0 × 10^{9} - 4.8 × 10^{11} CFU	3- 12	yes	Random	BFM, WMD	-0.42	-1.08, 0.23	Non-sig.	83.6	< 0.001	⊕⊕⊕◯ Moderate
												BF%, WMD	-0.6	-1.2, -0.01	Sig.	18.7	0.295	⊕⊕⊕◯ Moderate
Dong et al. 2019	DBRCTs	MetS	1,544	NR	> 12	18	Probiotics/control	10^{7}-10^{11} CFU/g	3-24	no	Random	BFM, SMD	–0.31	–0.64, 0.03	0.07	69	0.003	⊕⊕◯◯ Low
												BF%, SMD	–0.30	–0.58, –0.02	0.04	55	0.05	⊕⊕◯◯ Low
John et al, 2018	DBRCT	Overweight, obese	NR	NR	NR	21	Probiotic/placebo	0.007 × 109-150 ×109	2–24	yes	Random	BFM, MD	-0.94	-1.17, -0.72	<0.00001	0	0.91	⊕⊕◯◯ Low
							Prebiotic/placebo					BFM, MD	0.01	-0.58, 0.61	0.97	30	0.24	⊕⊕◯◯ Low

Koutnikova et al. 2019	RCT	Obesity, T2DM, NAFLD	6826	NR	NR	105	Probiotics/ control	10^{7} - 10^{12} CFU	2–28	yes	Random	BFM, MD	−0.62	−0.91 ,−0.34	NR	16.3	NR	⊕⊕⊕◯ Moderate
Mohammadi et al. 2019	RCT	Overweight, obese children and adolescents	410	B	7-16	9	Pro-/synbiotics Vs.Placebo	2.0× 10^{8}- 10^{10}	4-16	yes	Random	BF%, WMD	0.41	-1.70, 2.51	0.70	78.1	0.003	⊕⊕⊕◯ Moderate
Wang et al., 2019	RCT	Healthy obese, overweight	821	B	NR	12	Probiotic/control	$1×10^{7}$- $2×10^{11}$ CFU	8-24	yes	Random	BFM, MD	−0.91	−1.19, −0.63	<0.00001	43	0.08	⊕⊕⊕◯ Moderate
Cao et al. 2020	RCT	Overweight/ obese	2105	B	18–85	32	Probiotic or symbiotic/ placebo	10^{8}- 1.5× 10^{11}	3-12	yes	Random	BF%, WMD	-0.75	−0.90, −0.61	NR (Sig.)	63.7	0.001	⊕⊕⊕◯ Moderate
Companys et al. 2020	RCTs and observational study	obesity/overweight, T2DM, hypercholesterolemia, MetS	1,324,640	NR	18 - 75	37 RCT+ 18	Probiotic supplementation into a dairy matrix /control	1× 10^{4} - 27 × 10^{10} CFU/d	6.4-24	yes	Random	BF%, WMD	−0.41	−0.6, −0.21	<0.001	67.5	0.015	⊕⊕⊕◯ Moderate
							Probiotic with powder or capsules/control					BFM, WMD	−0.30	−0.48, −0.12	0.001	49	0.047	⊕⊕⊕◯ Moderate
Hadi et al. 2020	RCT	Overweight, obesity	1357	B	25 - 77	23	Synbiotic/placebo	$1×10^{7}$- $2×10^{11}$	4 - 28	yes	Random	BF, WMD	0.25	-1.27, 1.78	0.74	67.3	0.009	⊕⊕◯◯ Low
Mohammadi et al. 2021	DBRCT	Overweight, obesity, hypertensive, DM	1519	B	35-80	19	Probiotics fermented	4.97 × 10^{6}-7 × 10^{11}	2-12	yes	Random	BF%, WMD	-3.75	-9.39, 1.9	0.193	90.3	< 0.001	⊕⊕⊕◯ Moderate

							milk products /placebo											
Pontes et al. 2021	RCT	Overweight, obese	1720	B	31-66	26	Probiotic/placebo	2×10^7 - 112.5 $\times 10^9$ CFU/d	3-24	yes	Random	BFM, MD	−0.71	−1.1, −0.32	0.0004	55	0.009	⊕⊕◯◯ Low
												BF%, MD	-0.66	-1.05, -0.27	0.001	46	0.07	⊕⊕◯◯ Low
Qu et al. 2021	RCT	Overweight, Obese	535	B	10-70	12	Prebiotic/ placebo	2-21 g/d	2- 25.7	yes	Random	BFM, WMD	0.11	−0.04, 0.25	0.15	NR	NR	⊕⊕◯◯ Low
Daghmouri et al. 2022	RCT	Morbid obesity under bariatric surgery	279	NR	34-45	5	Probiotics/placebo	1×10^8 -1 $\times 10^{10}$ CFU/g	12-24	yes	Random	BFM, MD	2.46	-3.74, 8.66	0.44	91	< 0.01	⊕◯◯◯ Very Low
Li Z et al. 2022	RCT	PostmeNopausal with Overweight, obese	281	F	53-62	6	Probiotics/control	2.5 $\times 10^9$ -9.4 $\times 10^{10}$ CFU/d	5-12	yes	Random	BFM, MD	-0.43	-4.35, 3.48	Non-sig.	0	1	⊕⊕⊕◯ Moderate
Peckmezian et al. 2022	RCT	Overweight with/without major health condition	309	B	41.7 ± 8.9	11	Probiotics/control	1.79 x 10^6-2 x 10^{10}	3- 28	yes	Random	BFM, MD	−0.61	−0.77, −0.45	<0.001	0	1	⊕⊕⊕◯ Moderate
			231			10	Synbiotics/control					BFM, MD	−1.53	−2.95, −0.12	0.034	0	0.98	⊕⊕⊕◯ Moderate
Zhu et al., 2022	RCT	Obese	1818	B	47.89	32	Probiotic/placebo	NR	NR	NR	Random	BF%, MD	-0.82	-1.04, -0.61	<0.01	NR	NR	⊕⊕⊕⊕ High
Thu et al., 2023	RCT	Breast cancer	571	F	18-75	13	Probiotics/control	Varied	2–10	yes	Random	BF%, MD	−10.18	−32.26, 11.90	0.37	99	<0.00001	⊕⊕⊕◯ Moderate

Zhou et al, 2023	RCT	NAFLD	1037	B	10-64	21	Probiotic/control	NR	8-56	yes	Fixed	BFM, MD	−1.20	−3.29, 0.88	0.26	0	0.5	⊕⊕⊕◯ Moderate

Legend: B: Both; **F:** Female; **M:** Male; **ROB:** Risk of Bias; **NR:** not reported; **MA:** meta-analysis; **RCTs:** randomized controlled trials; **DBRCTs:** double-blind randomized controlled trials; **SBRCTs:** single-blind randomized controlled trials, **WMD:** Weighted mean difference; **SMD:** Standardized mean difference; **ASMRT:** A MeaSurement Tool to Assess systematic Reviews; **T2DM:** type 2 diabetes mellitus; **PCOS:** Polycystic ovary syndrome; **Sig.:** Significant; **BFM:** Body fat mass; **BF%:** Body fat percent; **NAFLD:** Non-alcoholic fatty liver disease; **PM:** post-menopausal; **GDM:** gestational diabetes mellitus; **MetS:** Metabolic syndrome; **%EWL:** Percentage of excess weight loss; **HC:** Hip circumference; **WHR:** Waist to hip ratio; **GRADE:** Grading of Recommendations Assessment, Development, and Evaluation..
